# Prognostic Factors and Outcomes for Post-Radiation Sarcoma—A Systematic Review and Meta-Analysis

**DOI:** 10.3390/cancers18152414

**Published:** 2026-07-27

**Authors:** Melissa Harbrücker, Maximilian Moeller, Carla Krug, Svetlana Hetjens, Madelaine Hettler, Steffen Seyfried, Christoph Reissfelder, Bernd Kasper, Christoph Brochhausen, Stefan Fröhling, Veronica Teleanu, Philipp Ströbel, Ulrich Kneser, Peter Hohenberger, Jens Jakob

**Affiliations:** 1 Department of Surgery, University Medical Centre Mannheim, Medical Faculty Mannheim, Heidelberg University, 68135 Mannheim, Germany; 2 DKFZ Hector Cancer Institute, University Medical Center Mannheim, 69120 Heidelberg, Germany; 3 Medical Statistics, Biomathematics and Information Processing, Medical Faculty Mannheim, Heidelberg University, 68135 Mannheim, Germany; 4 Sarcoma Unit, University Medical Centre Mannheim, Medical Faculty Mannheim, Heidelberg University, 68135 Mannheim, Germany; 5 Institute of Pathology, University Medical Centre Mannheim, Medical Faculty Mannheim, Heidelberg University, 68135 Mannheim, Germany; 6 National Center for Tumor Diseases (NCT), NCT Heidelberg, a Partnership Between DKFZ and Heidelberg University Hospital, 69120 Heidelberg, Germany; 7 German Cancer Consortium (DKTK), Core Center Heidelberg, 69120 Heidelberg, Germany; 8 Division on Translational Precision Medicine, Institute of Human Genetics, Heidelberg University, 69120 Heidelberg, Germany; 9 Division of Translational Medical Oncology, German Cancer Research Center (DKFZ), 69120 Heidelberg, Germany; 10 Institute of Pathology, University Medical Center Göttingen, University of Göttingen, 37075 Göttingen, Germany; 11 Department of Hand, Plastic and Reconstructive Surgery, Microsurgery, Burn Trauma Center, BG Trauma Center Ludwigshafen, University of Heidelberg, 67071 Ludwigshafen, Germany; 12 Division of Surgical Oncology and Thoracic Surgery, University Medical Centre Mannheim, Medical Faculty Mannheim, Heidelberg University, 68135 Mannheim, Germany; 13 Sarcoma Unit, Department of Surgery, University Medical Center, Medical Faculty Mannheim, Heidelberg University, 68135 Mannheim, Germany

**Keywords:** post-radiation sarcoma, radiation-induced sarcoma, secondary malignancy, angiosarcoma, prognostic factors

## Abstract

**Background/Objectives:** Post-radiation sarcomas (PRS) are secondary malignancies occurring years after radiotherapy. Due to low incidence, evidence on prognostic factors and management is limited. **Methods:** MEDLINE, Embase, and the Cochrane Library were searched from January 1960 to January 2026. Eligible studies included observational cohorts reporting outcomes of histologically confirmed PRS. We excluded case reports, case series with fewer than ten patients, reviews, editorials, and studies without survival or recurrence outcomes. Two authors independently screened studies, extracted data, and assessed risk of bias. Hazard ratios (HRs) and 95% confidence intervals were extracted or calculated. The primary outcome was overall survival. Heterogeneity was assessed using the I^2^ statistic. This study was registered with PROSPERO (CRD42023457401). **Results:** The search identified 4222 records, of which 28 studies comprising 2438 patients were eligible for analysis. Most patients were female (2008, 83.3%), reflecting a predominance of breast radiation-associated angiosarcoma. Older age, larger tumor size, higher histological grade, and positive surgical margins were consistently associated with worse overall survival. Complete (R0) resection was associated with improved survival (pooled HR < 1.0), as was treatment in specialized sarcoma centers. Chemotherapy and additional radiotherapy were not associated with improved overall survival. Substantial heterogeneity was observed (I^2^ > 50% for several analyses). Overall risk of bias was moderate. **Conclusions:** PRS share key prognostic determinants with sporadic sarcomas. Complete surgical resection and management in specialized sarcoma centers are the most important factors associated with improved outcomes. These findings support centralized care and highlight the need for prospective research to improve risk stratification and management of PRS.

## Simple Summary

Radiation therapy is an important part of cancer treatment, but rarely a sarcoma can develop years later in the irradiated area. Because post-radiation sarcomas are uncommon, prognostic factors and optimal treatment remain uncertain. We summarized 28 studies including 2438 patients. Older age, larger tumor size, higher tumor grade, and incomplete tumor removal were associated with worse outcomes, similar to sporadic sarcomas. The grading finding is clinically relevant because these tumors have often been considered uniformly high-grade; our results suggest that meaningful grade differences still exist and may help identify higher-risk patients. Complete surgical resection and treatment in specialized sarcoma centers were the most important modifiable factors linked to better outcomes. Evidence for chemotherapy, radiotherapy, and other multimodal treatments remains limited, although selected patients may benefit from neoadjuvant systemic therapy.

## 1. Introduction

Post-radiation sarcomas (PRS) are rare malignancies and constitute a distinct clinical and therapeutic challenge. They are generally considered to arise in previously irradiated fields following radiotherapy for a prior malignancy; however, a definitive causal relationship between radiotherapy and the development of PRS has not been conclusively established to date. The evidence base on PRS remains limited, and both etiological co-factors and treatment-related outcomes are insufficiently characterized. PRS might also develop after radiotherapy for non-malignant diseases like desmoid tumors [1,2,3].

The incidence of PRS is estimated to be very low, approximately 0.03–0.3% of all irradiated patients, accounting for approximately 2.5–5.5% of all sarcomas [4,5,6,7,8]; and only limited data are available from epidemiological studies and cancer registries. A few initiatives such as CANSARCC [9] illustrate structured efforts to collect clinical data on PRS to explore potential familial predisposition. While hereditary cancer syndromes, including Li–Fraumeni syndrome [10] or neurofibromatosis, represent a plausible and documented risk factor in several cases, they are unlikely to account for the majority of PRS. Additional co-factors have been reported, including chemotherapy administered for the first malignancy—particularly anthracycline-based regimens [11]—as well as radiotherapy-related parameters such as dose and treatment technique [12,13].

Treatment of PRS generally follows principles established for other sarcomas, although prior irradiation substantially constrains standard therapeutic approaches. Surgical resection remains the cornerstone of treatment, but increased radicality [8,14] has been proposed to achieve local control. The role of perioperative radiotherapy is markedly limited by cumulative toxicity and concerns regarding the induction of further radiation-associated malignancies. Systemic chemotherapy can be effective in selected patients; notably, recent reports in post-radiation angiosarcoma suggest activity of taxane-based regimens [15], despite their non-standard role in first-line sarcoma treatment. Treatment decisions may further be influenced by tumor-specific factors such as tumor size, which is especially difficult to quantify in post-radiation angiosarcoma, which are often multifocal. Most PRS are classified high grade lesions. 

Given the rarity of PRS and the heterogeneity of PRS-cohorts published recently, individual studies are frequently not sufficient to evaluate risk modifiers and treatment effects. A systematic review with meta-analysis is warranted to summarize and affirm available evidence. This review was prospectively registered in PROSPERO (CRD42023457401). The primary objectives were to assess the reporting and impact of potential co-factors and treatment-related variables in published PRS cohorts regarding treatment objectives. Outcome data were intended to be pooled, when feasible, to derive estimates and to foster future research efforts.

## 2. Materials and Methods

### 2.1. Study Design and Registration

We conducted a systematic review and meta-analysis in accordance with the PRISMA 2020 guidelines; the PRISMA checklist is provided in Table S5. 

### 2.2. Search Strategy and Selection Criteria

We searched MEDLINE (via PubMed), Embase, and the Cochrane Library from 1 January 1960 to November 2023, with an update on 13 January 2026. The search strategy combined controlled vocabulary and free-text terms for sarcoma and radiation-induced neoplasms (full strategy in Table S4). No date or language restrictions were applied at the search stage.

Eligible studies were observational cohort studies reporting outcomes of adult patients with histologically confirmed post-radiation sarcoma defined according to modified Cahan’s criteria. We included studies reporting hazard ratios (HRs) for survival or recurrence outcomes, or providing sufficient data to reconstruct HRs.

We excluded case reports, case series with fewer than ten patients, pediatric-only cohorts, preclinical studies, reviews, editorials, non–peer-reviewed publications, and studies without survival endpoints.

Two authors (MH, CK) independently screened titles, abstracts, and full texts. Disagreements were resolved by a third author (JJ).

### 2.3. Data Extraction and Definitions

Two authors (MM, CK) independently extracted study-level summary data. Individual patient-level data were not requested.

Extracted variables included study characteristics, demographics, primary tumor, radiation exposure, latency, sarcoma subtype, treatment variables, and reported hazard ratio with 95% confidence interval (HR [95% CI]) for:

- Overall survival (OS)
- Disease-specific survival (DSS)
- Distant recurrence-free survival (DRFS)
- Local recurrence-free survival (LRFS)

Distant recurrence-free survival (DRFS), distant metastasis-free survival (DMFS), metastasis-free survival (MFS), and time to distant progression (TTDP) were harmonized as distant recurrence-free survival (DRFS). Local recurrence-free survival (LRFS) and time to local progression (TTLP) were harmonized as LRFS (TTLP restricted to R0 resections).

For age and tumor size, study-defined cutoffs were retained. “High” versus “low” categories were pooled without cross-study harmonization. Where age directionality was ambiguous [16], the variable was excluded from quantitative synthesis.

Grading was harmonized to G1/2 versus G3 where possible. Margin status was harmonized to R0, R1, and R2 when reported; otherwise it was negative versus positive.

Sex was extracted and reported as a descriptive variable. Gender was not separately defined or analyzed, as this information was not available in the included studies. 

When HRs were not reported, we reconstructed estimates using the Tierney method [17] based on published data or Kaplan–Meier curves when extractable. Where sufficient event data were available, Cox regression was performed to derive HRs.

### 2.4. Risk of Bias Assessment

Risk of bias was assessed using the Newcastle–Ottawa Scale (NOS). Two authors (MM, CK) independently performed assessment; disagreements were resolved by consensus. Scores range from 0 to 9, with higher scores indicating higher methodological quality.

### 2.5. Statistical Analysis

Meta-analyses were conducted using Review Manager (RevMan) 5.4.

HRs were pooled using the generic inverse variance method. Heterogeneity was quantified using I^2^. Fixed-effects models were used unless heterogeneity was substantial (I^2^ > 50% or χ^2^ *p* < 0.1), in which case random-effects models (DerSimonian–Laird) were applied.

Publication bias was assessed by funnel plot and Egger’s test. Since standard methods for assessing publication bias require at least 10 studies [18], quantitative analysis was only feasible for grading (OS), where nine studies (13 data points) were available. Publication bias could not be assessed for other risk factors due to the limited number of studies.

Primary analyses combined univariate and multivariate HRs. Sensitivity analyses were restricted to multivariate HRs. If only univariate HRs were available, sensitivity analyses were limited to studies with NOS ≥ 7 and excluded reconstructed HRs.

Predefined subgroup analyses included margin category, size thresholds, age cutoffs, histology (angiosarcoma), and perioperative treatment setting.

### 2.6. Role of the Funding Source

The funder of the study had no role in study design, data collection, data analysis, data interpretation, or writing of the report.

## 3. Results

**Figure 1:**
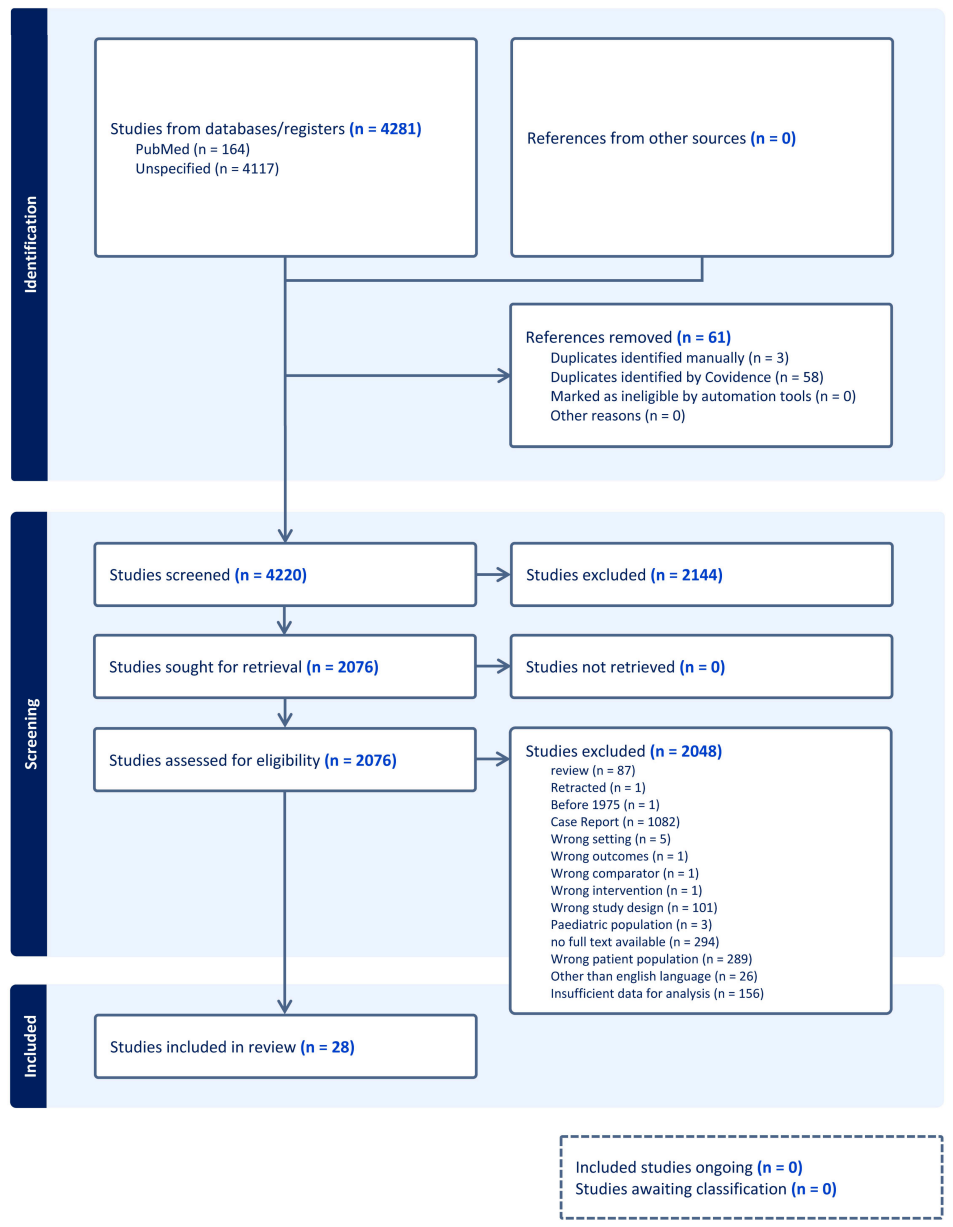
PRISMA diagram to illustrate the screening process.

The search yielded 4281 records; after removal of 59 duplicates, 4222 studies were screened and 2078 full texts assessed. Twenty-eight retrospective cohort studies were included (Figure 1). 

All studies were published after 2004, with 21 of the 28 studies published within the past ten years. Overall, 2855 patients were reported, of whom 2438 were eligible for quantitative synthesis. Sex distribution was available for most cohorts (2008 women, 402 men; two studies did not report sex). All but one study were of moderate to high quality (NOS ≥ 5; Table 1 and Table S1).

**Figure 2:**
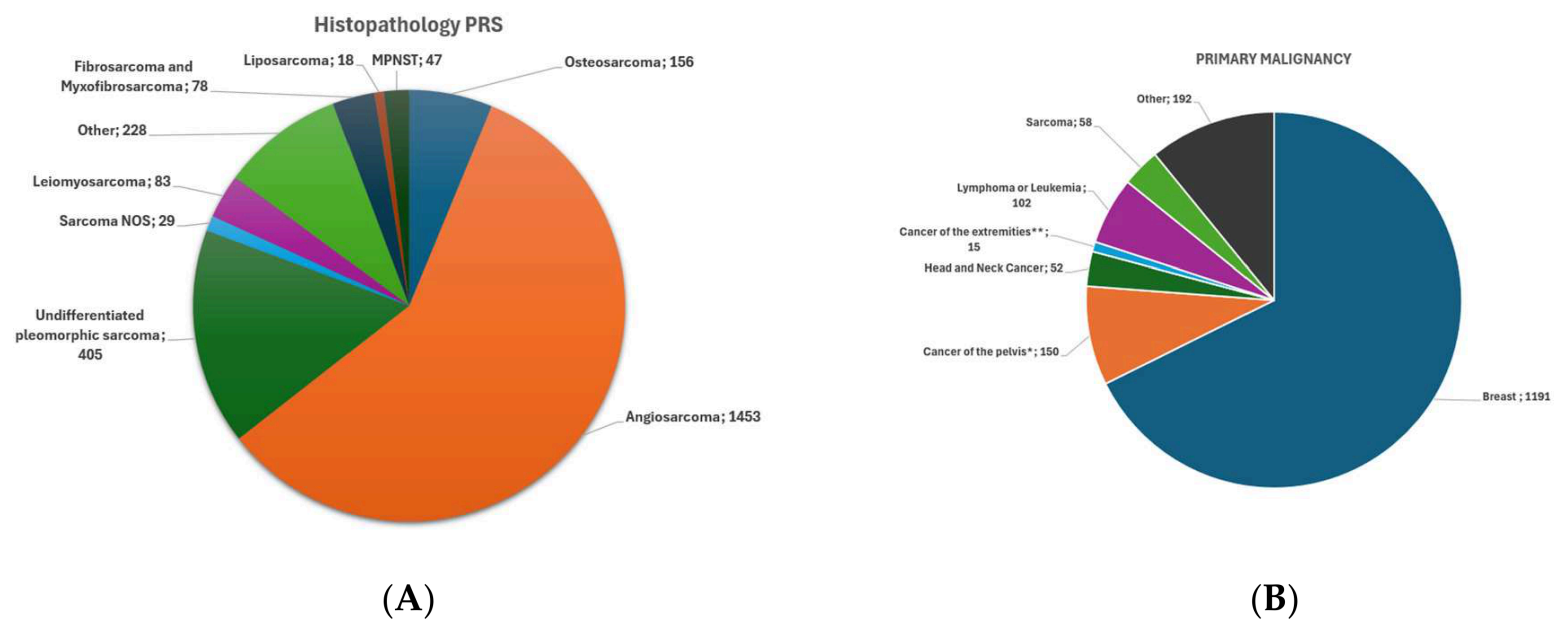
(**A**) Representation of the PRS Histology distribution of the pooled cohort (malignant fibrous histiocytoma was subsumed under Undifferentiated Pleomorphic Sarcoma (UPS)), Malignant Peripheral Nerve Sheath Tumor (MPNST), Not Otherwise Specified (NOS). (**B**) Primary malignant disease of the patient cohort; * including genitourinary tract cancers (e.g., *n* = 34 prostate cancer); ** excluding sarcoma; Others = Wilms tumor, thyroid cancer, non-small-cell lung cancer, leukemia, retinoblastoma, cutaneous squamous cell carcinoma, thymoma, congenital lymphovascular malformations, mucoepidermoid carcinoma, chordoma, medulloblastoma, ependymoma, craniopharyngioma, anaplastic astrocytoma, oligodendroglioma, meningioma, acute lymphoblastic leukemia, adenoid cystic carcinoma, embryonal rhabdomyosarcoma, CNS cancer, genetic predisposition, benign tumor, giant cell tumor of the bone, ovarian teratoma, unknown, (added by authors: gastrointestinal (*n* = 9), skin (*n* = 4), colorectal (*n* = 5).

Fourteen studies exclusively included angiosarcoma (Kuba et al. provided HRs only for angiosarcoma); three included only soft tissue or bone sarcoma; eight included both (Table 1). Angiosarcoma accounted for 1453 patients (58%), followed by undifferentiated pleomorphic sarcoma (405 patients, 16%) (Figure 2A). Among 25 studies reporting the primary malignancy, breast cancer was most common (1191 patients, 68%) (Figure 2B). 

Median radiation dose documented was most frequently 50 Gy (range 10.8–160 Gy). Latency ranged from 0.5 to 74 years, with a median of 7 years most frequently reported. Median age at PRS diagnosis was 65–70 years (Table 1).

Pooled analyses are summarized in Table 2 and Table S6; corresponding forest plots are shown in Figures S1–S8.

Older age was associated with worse OS (HR 1.85 [95% CI 1.28–2.69]; I^2^ = 58%) and DSS (HR 1.16 [95% CI 1.07–1.27]; I^2^ = 46%) (Figure S1a,b). Continuous age analyses were not significant (Table S6; Figure S1c–e).

Tumor size was associated with worse OS (HR 1.54 [95% CI 1.02–2.35]; I^2^ = 73%) and DSS (HR 2.06 [95% CI 1.34–3.18]; I^2^ = 24%) (Table 2 and Table S6; Figure S2a,b). Size as a continuous variable yielded HR 1.13 [95% CI 1.05–1.21] for OS (Figure S2c). No significant association was observed for LRFS or DRFS (Table 1, Figure S2d,e).

**Figure 3:**
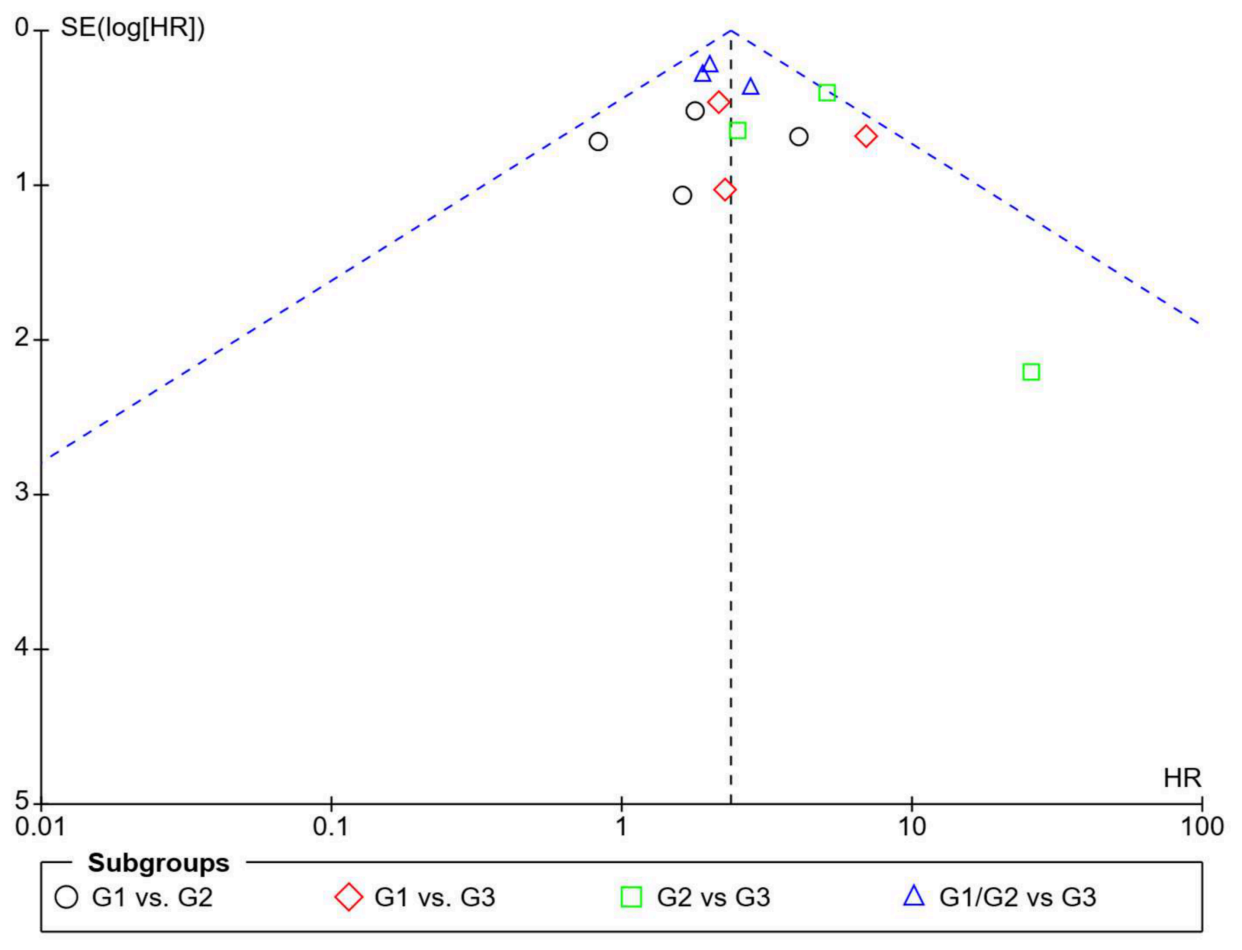
Evaluation of publication bias using the funnel plot for the risk factor grading.

Higher histologic grade was associated with worse OS (HR 2.38 [95% CI 1.86–3.04]; I^2^ = 0%) (Figure S3a). Ten studies provided data on tumor grading, comprising 90 patients with G1, 287 with G2, and 748 with G3 tumors. A detailed overview is provided in Table S2. No evidence of publication bias was observed for this analysis (Egger’s test *p* = 0.14; Figure 3). Subgroup analyses showed HR 3.00 [95% CI 1.49–6.07] for G1 versus G3, HR 4.37 [95% CI 2.26–8.46] for G2 versus G3, and HR 2.10 [95% CI 1.53–2.88] for G1/G2 versus G3 (Table S6). For DRFS, pooled HR was 2.60 [95% CI 1.65–4.09]; I^2^ = 3% (Figure S3b).

Multifocal versus single-lesion disease showed no difference in OS (HR 1.01 [95% CI 0.70–1.45]; I^2^ = 0%) (Figure S4a). Deep tumor location compared with superficial location was associated with worse OS (HR 2.58 [95% CI 1.70–3.90]; I^2^ = 0%) (Figure S4b).

Treatment in multidisciplinary sarcoma centers was associated with improved DSS (HR 0.27 [95% CI 0.14–0.53]; I^2^ = 0%) (Figure S5).

**Figure 4:**
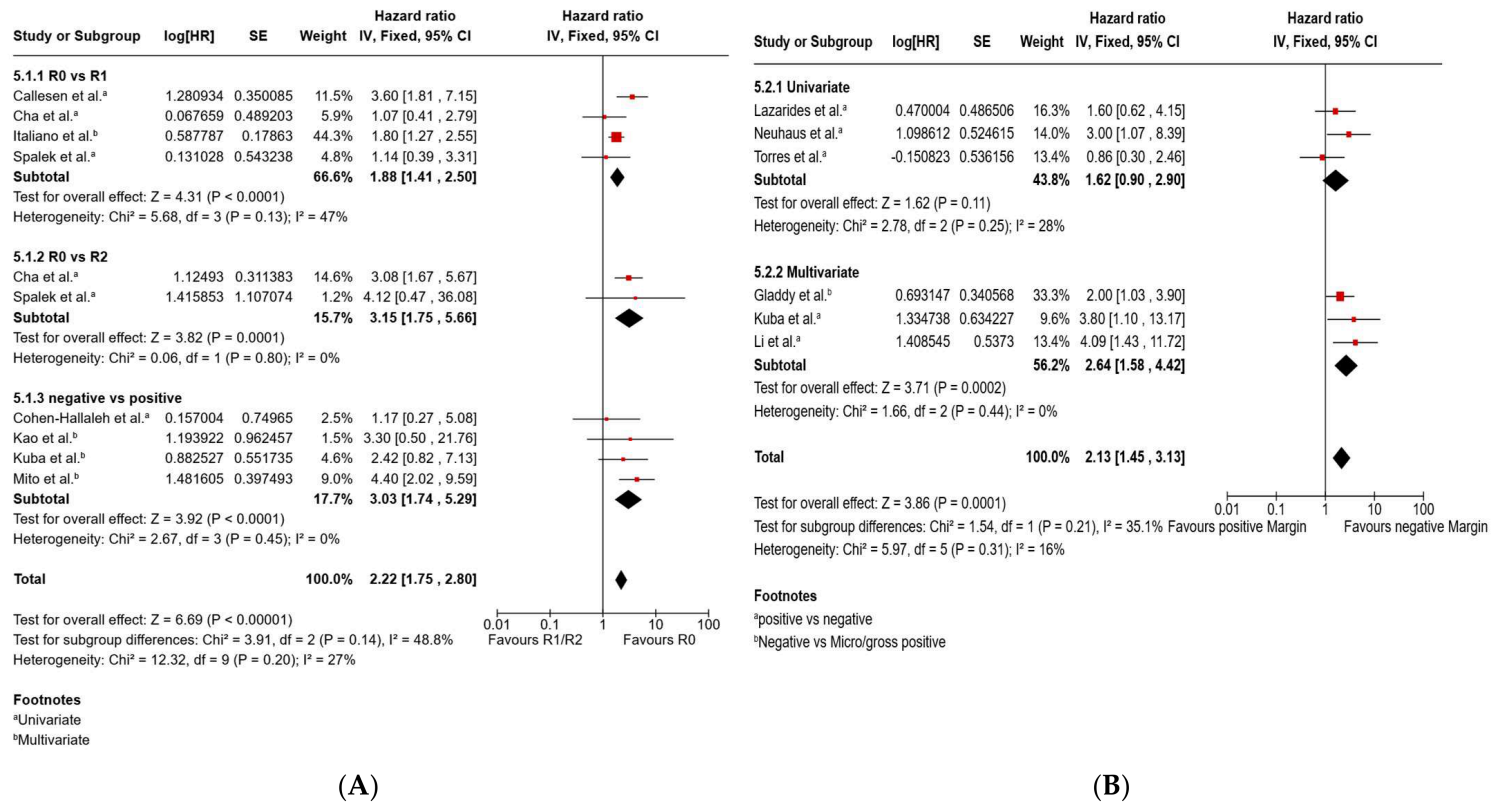

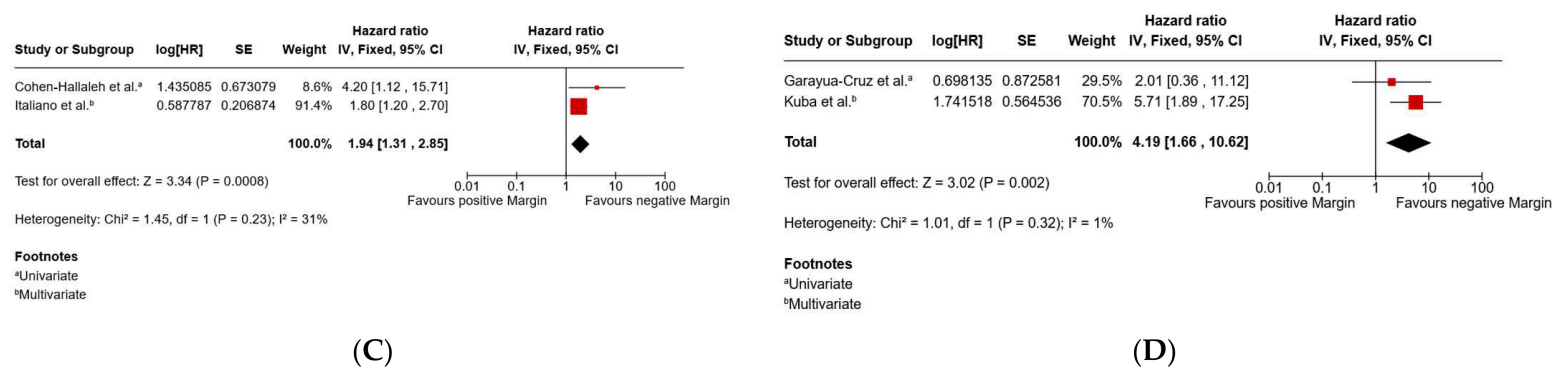
Forest plots surgical margin: (**A**) OS [14,22,25,26,27,31,33,41], (**B**) DSS [24,25,34,37,39,40], (**C**) DRFS (negative vs. positive) [31,33], (**D**) LRFS (negative vs. positive) [20,25].

Positive surgical margins were consistently associated with worse outcomes (Figure 4A–D). For OS, pooled HR was 2.22 [95% CI 1.75–2.80]; I^2^ = 27% (Figure 4A). For DSS, HR was 2.13 [95% CI 1.45–3.13]; I^2^ = 16% (Figure 4B). For LRFS, HR was 4.19 [95% CI 1.66–10.62]; I^2^ = 1%, and for DRFS 1.94 [95% CI 1.31–2.85]; I^2^ = 31% (Figure 4C,D). Subgroup analyses for OS showed HR 1.88 [95% CI 1.41–2.50] for R0 versus R1 and HR 3.15 [95% CI 1.75–5.66] for R0 versus R2 resections; negative versus positive margins yielded HR 3.03 [95% CI 1.74–5.29] (Table S6).

Radical versus limited resection showed no significant OS difference (HR 0.54 [95% CI 0.23–1.22]; I^2^ = 57%) (Figure S6a), but was associated with improved DSS (HR 0.53 [95% CI 0.30–0.94]; I^2^ = 20%) (Figure S6b) and improved LRFS (HR 0.43 [95% CI 0.19–0.94]; I^2^ = 49%) (Figure S6c). Surgery versus no surgery yielded HR 4.37 [95% CI 1.34–14.25]; I^2^ = 88% (Figure S6d).

Radiotherapy for PRS at any time was not associated with improved overall survival (HR 1.37 [95% CI 0.94–1.98]; I^2^ = 32%) (Figure S7a). One study reported multivariate HR 1.77 [95% CI 1.01–3.11]. In subgroup analyses, adjuvant radiotherapy showed HR 1.91 [95% CI 1.11–3.29], whereas mixed neo-/adjuvant regimens (HR 1.02 [95% CI 0.62–1.70]) were not significant (Table S6). No significant effect was observed for LRFS (Figure S7b).

Chemotherapy for PRS at any time was not associated with improved overall survival (HR 0.82 [95% CI 0.60–1.12]; I^2^ = 31%) (Figure S8a). The systemic treatment regimens and agents used across the included studies are summarized in Table S3. Subgroup analyses showed no significant OS effect for mixed (HR 0.88 [95% CI 0.58–1.32]), adjuvant (HR 2.17 [95% CI 0.60–7.80]), or neoadjuvant chemotherapy (HR 0.65 [95% CI 0.39–1.07]) (Table S6). For DSS, pooled HR was 0.85 [95% CI 0.33–2.20]; I^2^ = 73% (Figure S8b). Neoadjuvant chemotherapy showed HR 0.41 [95% CI 0.19–0.90], whereas mixed regimens (HR 1.18 [95% CI 0.33–4.18]) were not significant (Table S6). For LRFS, pooled HR was 0.45 [95% CI 0.31–0.66]; I^2^ = 0% (Figure S8c). For DRFS, pooled HR was 1.06 [95% CI 0.59–1.93]; I^2^ = 0% (Figure S8d).

Factors that could not be pooled, as well as those with limited or partially poolable data, including sex, reconstructive surgery, and tumor localization, are summarized narratively in the Supplementary Materials.

## 4. Discussion

Post-radiation sarcomas (PRS) have been recognized for decades; however, evidence to guide their optimal management remains limited. All of the 28 studies included in this meta-analysis were retrospective cohort studies. The analysis spans a relatively long time period, but the majority of included studies were published in recent years, with 21 of 28 studies (75%) originating from the past decade and none predating 2004. More than 1000 publications were excluded because they were case reports, underscoring both the rare nature of PRS and the methodological limitations inherent to this field. To our knowledge, this is the first quantitative meta-analysis synthesizing prognostic and treatment-related risk factors in PRS. The exclusion of case reports and very small case series (<10 patients), although methodologically necessary, highlights a structural limitation in rare diseases: much of the literature does not contribute to generate evidence.

Across the pooled cohort (*n* = 2438 eligible patients), classical sarcoma parameters retained robust prognostic value. Larger tumor size (OS HR 1.54 [95% CI 1.02–2.35]; DSS HR 2.06 [95% CI 1.34–3.18]) and higher histological grade (OS HR 2.38 [95% CI 1.86–3.04]; DRFS HR 2.60 [95% CI 1.65–4.09]) were consistently associated with worse outcomes. The graded effect across G1 vs. G3 (HR 3.00 [95% CI 1.49–6.07]) and G2 vs. G3 (HR 4.37 [95% CI 2.26–8.46]) confirms the validity of established grading systems such as Fédération nationale des centres de lutte contre le cancer (FNCLCC), even in PRS cohorts (G1 vs. G3 [9,26,35]; G2 vs. G3 [22,27,28]). Although grading criteria have evolved over time, the prognostic consistency across decades suggests that fundamental pathological determinants—mitotic rate, necrosis, and differentiation—remain biologically relevant. Age was likewise associated with inferior overall survival (HR 1.85 [95% CI 1.28–2.69]), consistent with sarcoma prognostic tools such as Sarculator [42] and PERSARC [43]. These findings indicate that PRS follow the same core biological risk architecture as sporadic sarcomas.

Surgical margins emerged as the most consistent prognostic determinant across all outcomes (OS HR 2.22 [1.75–2.80]; DSS HR 2.13 [1.45–3.13]; LRFS HR 4.19 [1.66–10.62]; DRFS HR 1.94 [1.31–2.85]). The effect was robust across R0 vs. R1 and R0 vs. R2 comparisons. Radical surgery improved DSS (HR 0.53 [0.30–0.94]; [16,24,34]) and LRFS (HR 0.43 [0.19–0.94]; [28,29]). These data strongly support wide en bloc resection, ideally encompassing the irradiated field. Notably, no study systematically incorporated original radiation plans to correlate margin status with prior dose distribution. Furthermore, no study ever evaluated whether resection of the radiation field results in better disease-free survival rates and looked into the reconstructive measures required to cover resulting soft tissue defects. The absence of outcome differences between multifocal and unifocal disease (HR 1.01 [0.70–1.45]; [19,21,23]) supports the hypothesis that multifocal presentations may reflect contiguous field involvement rather than biologically distinct tumor behavior, reinforcing tumor burden rather than lesion count as the relevant prognostic variable.

Analysis of perioperative therapies requires careful interpretation. Additional radiotherapy was not associated with improved overall survival (HR 1.37 [0.94–1.98] [21,22,26,28,35]). In adjuvant settings, radiotherapy did not demonstrate a survival benefit in pooled analyses (HR 1.91 [1.11–3.29]; [28,35]), a finding that should be interpreted with caution given potential confounding by disease severity and indication bias. This likely reflects selection bias: patients receiving re-irradiation are often those with suboptimal margins or locally advanced disease. Moreover, cumulative radiation toxicity limits therapeutic intensity. No prospective cohort has evaluated preoperative versus postoperative radiotherapy in PRS. In sporadic sarcoma, preoperative radiotherapy has shown advantages in selected settings [44]; however, these data cannot be extrapolated directly. In PRS, postoperative radiotherapy appears primarily salvage-oriented rather than strategy-driven.

Chemotherapy showed no overall survival benefit (OS HR 0.82 [0.60–1.12]; DSS HR 0.85 [0.33–2.20]). Subgroup analyses suggested a potential advantage for neoadjuvant chemotherapy in DSS (HR 0.41 [0.19–0.90]; [16]), whereas adjuvant chemotherapy showed no benefit and in some subgroups trended unfavorably (OS HR 2.17 [0.60–7.80] [28]). This again likely reflects selection bias: neoadjuvant treatment is more frequently delivered in specialized centers and in planned multimodal concepts, whereas adjuvant therapy may represent escalation in biologically aggressive cases. Only one study [21] analyzed quantitatively detailed chemotherapy regimens, with substantial heterogeneity. Many patients had prior anthracycline exposure for their primary malignancy, limiting the feasibility of re-challenge. Observational data demonstrate improved outcomes for patients treated in specialized sarcoma centers [45], where structured multimodal treatment strategies are more consistently implemented. It is plausible that neoadjuvant chemotherapy, when delivered within expert settings, is more frequently planned and protocol-driven rather than administered as escalation therapy. In line with this, van der Burg et al. [15] reported improved oncological outcomes with neoadjuvant taxane-based chemotherapy in radiation-associated angiosarcoma. Although based on a small cohort, the study provides a coherent treatment rationale and has informed multimodal treatment pathways. Accordingly, chemotherapy and pre- or postoperative radiation therapy may be considered as part of an individualized, multidisciplinary approach, particularly if they can improve complete resection or enhance locoregional control. Given the strong prognostic significance of the resection margin status, complex or initially inoperable cases should be evaluated at experienced multidisciplinary sarcoma centers.

Several cohorts analyzed bone and soft tissue sarcoma together [9,14,22,26,27,40], and despite lineage-specific protocols in sporadic sarcoma (e.g., osteosarcoma or Ewing regimens), margin status, tumor size, grade, and age consistently determined outcome in PRS. In clinical practice bone and soft-tissue PRS are treated according to distinct algorithms; however, pooled analyses demonstrate similar prognostic patterns across entities. This convergence supports a unifying framework: irrespective of histological subtype, PRS behave as sarcomas arising in irradiated tissue, where local control predominates. Excluding bone sarcomas from the present analysis would have further reduced the already limited evidence base, underscoring the structural challenge in this rare disease, as outlined above.

Importantly, this meta-analysis does not address pathogenesis. Data on genetic susceptibility and risk factors for the development of PRS remain scarce. Although our systematic search identified several studies describing molecular alterations in radiation-associated sarcomas, these analyses were not linked to survival outcomes and were therefore beyond the scope of this quantitative synthesis; they will be examined separately in a translational framework. Outcome-focused studies such as CanSaRCC provide valuable clinical data but are rarely integrated with systematic molecular characterization [9], highlighting a persistent gap between clinical and translational research. Future studies could also investigate whether molecular profiling of surgical specimens can inform treatment selection and whether longitudinal analysis of circulating tumor DNA can help detect residual molecular disease or metastatic recurrence. Multi-institutional observations, including the study by Zhang et al. [11], suggest that prior chemotherapy exposure may shorten the latency period to PRS onset, raising the possibility that systemic therapy acts as a modifying factor in tumorigenesis. However, this hypothesis remains insufficiently validated. Large-scale cancer registry analyses and prospective cohort initiatives will be essential to disentangle treatment-related influences from intrinsic biological susceptibility.

Clinical heterogeneity remained an important limitation. The included cohorts differed regarding demographics, primary malignancy, sarcoma subtype and location, previous radiation exposure, latency, treatment era, and therapeutic management. Although clinically comparable contrasts were pooled and subgroup and sensitivity analyses were performed, residual heterogeneity and study-level confounding could not be excluded. Given the low incidence of this rare disease, a large prospective trial to validate the described risk factors and evaluate additional risk factors is unlikely. For this reason, multicenter and interdisciplinary observational studies such as TTRIS-CSI are necessary for further research into this rare disease.

In the absence of clearly defined causal mechanisms, terminology has gradually shifted from “radiation-induced” towards “radiation-associated” or “post-radiation” sarcoma, acknowledging both the multifactorial nature of oncogenesis and the current limitations in attributing strict causality; consequently, these terms were all incorporated into our search strategy.

## 5. Conclusions

The main findings of the study are

- post-radiation sarcomas share key prognostic factors with sporadic sarcomas such as size, histopathological grading, or complete surgical resection
- management in specialized sarcoma centers is the most important modifiable factor associated with improved outcome, and
- evidence for multimodal therapies remains limited while neoadjuvant systemic treatment may improve outcome.

These findings support centralization of care and prioritization of complete surgical resection whenever feasible.

## Figures and Tables

**Table 1:** Detailed Study characteristics.

Autor	Year	Country	Recruiting Period	No of Patients	No of Patients Analyzed	Sex	Sarcoma Type	Outcome of Interest	Risk Factors of Interest	Univariate/ Multivariate	Adjustment for Relevant Confounders	Median Age (Range)	Median Follow-Up	Median Initial Radiation Dose	Latency (Years) (Median Time to Secondary Malignancy Diagnosis)	Quality Assessment	Data Availability
Michot et al. [19]	2025	France	1988–2016	180	180	Female (*n* = 179) Male (*n* = 1)	Angiosarcoma of the breast	OS, DRFS, LRFS	Chemotherapy (neoadjuvant); Age (≥73 years); Tumor depth; Tumor size (≥ 45 cm); Tumor grade (G1/G2 vs. G3); Multifocality	univariate/multivariate	OS: age, tumor size, depth and grade DRFS: grade, chemotherapy LRFS: grade, age, chemotherapy	73 (43–90)	7 (0.9–24.1)	N/A	5.5(95% Cl: 4.2–6.2)	9/9	extracted
Garayua-Cruz et al. [20]	2024	USA	1986–2020	17	17	Female (*n* = 17) Male (*n* = 0)	Soft tissue sarcomas	DSS, LRFS	Surgical margin (negative vs. positive); Age (≥65 years); Chemotherapy (mixed cohort); Radiotherapy (mixed cohort)	univariate	N/A	65 (IQR 16)	6 (3–27)	57.9 Gy (IQR 12.9 Gy)	14 (IQR 10)	5/9	extracted
Palassini et al. [21]	2024	Italy	2000–2019	84	84	Female (*n* = 84) Male (*n* = 0)	Angiosarcoma of the breast	OS, RFS	Radiotherapy (mixed cohort); Chemotherapy (mixed cohort); Mulitfocal; Age (continuous), Size (continuous)	univariate	N/A	69 (26–87)	51 months (IQR 30–126)	N/A	7 (0.5–22)	9/9	extracted
van der Burg et al. [15]	2024	Netherlands	1994–2024	35	35	Female (*n* = 35) Male (*n* = 0)	Angiosarcoma of the breast	OS	Chemotherapy (Neoadjuvant)	univariate	N/A	mean age 64	41 months (21–84)	N/A	7 (5–10)	8/9	calculated
Ribeiro et al. [9]	2023	Canada	1996–2021	107	107	Female (*n* = 85) Male (*n* = 22)	Soft tissue and bone sarcomas	OS	Age (≥60 years); Tumor grade (G1 vs. G2 vs. G3); Tumor size (Maximum tumor diameter), sex, primary tumor site	multivariate	age, sex, maximum tumor diameter, time from RT to PRS diagnosis, PRS subtype, histologic grade, margin status, RT exposure, primary site	68.1 (18.5–89.6)	N/A	50 Gy (15–95)	12.9 (3–56.7)	7/9	extracted
Kao et al. [22]	2023	USA	1993–2018	94	94	Female (*n* = 40), Male (*n* = 54)	Soft tissue and bone sarcoma	OS	Age (>62 years); Margin (complete resection, incomplete resection, fragmented resection); Tumor Size (>6.5 cm); Chemotherapy (unspecified); Radiotherapy (unspecified); Histology; Tumor grade (intermediate vs. high)	univariate/multivariate	histology, grade, localization, margin, age, size, T stage	62 (10–87)	13 months (0–176)	N/A	10 (3–42)	9/9	extracted
Wong et al. [23]	2023	UK	2010–2022	24	22	Female (*n* = 22), Male (*n* = 0)	Angiosarcoma	OS	Age (>72); Multifocality; Tumor size (≥ 5 cm); Chemotherapy (mixed cohort)	univariate	N/A	65 (41–78)	66.5 months	40 Gy	6.5 (2.4–16.0)	6/9	extracted
Lazarides et al. [24]	2022	Canada	1985–2020	35	35 (Histology N = 33, Treatment N = 32)	Female (*n* = 15) Male (*n* = 20)	Bone sarcoma Pelvis and sacrum	DSS	Age (>40 years); Tumor size (>11 cm); Surgical margin (negative vs. positive); Chemotherapy (mixed cohort); Radiotherapy (mixed cohort); Resection strategy (radical (amputation) vs. limited (limb salvage)), sex, Location	univariate	N/A	57 (14–84)	95 months (20–383)	50 Gy (25–66 Gy)	12.7 (3–27)	9/9	extracted
Guram et al. [16]	2021	Canada	2004–2019	51	49	Female (*n* = 49) Male (*n* = 0)	Angiosarcoma of the breast	DSS	Tumor size (>11 cm); Surgical margin (negative vs. positive); Chemotherapy (Neoadjuvant); Resection strategy (radical vs. limited), Treatment in multidisciplinary sarcoma center	univariate/multivariate	age, initial treatment team, the use of neoadjuvant chemotherapy, location of initial treatment	74 (41–89)	26 months (4–182)	42.4 Gy (40–66 Gy)	8 (3–27)	7/9	extracted
Kuba et al. [25]	2021	USA	1998–2019 (Angiosarcoma) and 2014–2019 (other the Angiosarcoma)	99	81	Female (*n* = 81) Male (*n* = 0)	Angiosarcoma of the breast and soft tissue sarcoma	OS, LRFS, DRFS	Age (continuous); Surgical margin (negative vs. positive); Tumor size (continuous), Histology	multivariate	age, tumor size, margin status, MYC amplification	69 (48–95)	30 months (0–237)	N/A	7 (3–25)	8/9	extracted and calculated
Spalek et al. [26]	2021	Poland	1998–2019	58	58	Female (*n* = 43) Male (*n* = 15)	Soft tissue and bone sarcomas	OS	Tumor grade (G1 vs. G2 vs. G3); Radiotherapy (Peri-operative); Chemotherapy (mixed cohort); Surgical margin (R0 vs. R1 vs. R2), Location	univariate	N/A	57(20–84)	15 months (IQR 9–24)	N/A	11 (3–36)	7/9	extracted
Callesen et al. [14]	2020	Denmark	1979–2013	64	53	Female (*n* = 53) Male (*n* = 11)	Soft tissue and bone sarcomas	OS	Surgical margin (R0 vs. R1); Surgery (yes vs. no), Location	univariate	N/A	67 (29–82)	1.7 (0.1–34)	N/A	11 (3–36)	7/9	extracted
Mito et al. [27]	2019	USA	1994–2016	188	176	Female (*n* = 139) Male (*n* = 37)	Soft tissue and bone sarcomas	OS	Tumor grade (G1 vs. ≥G2); Surgical margin (negative vs. positive); Surgery (yes vs. no); Tumor depth	multivariate	N/A: potentially deep site, grade, surgery, margins	63 (27–88)	3.2 (0–16.6)	60 Gy (10.8–88.6)	11 (2–60)	9/9	extracted
Salminen et al. [28]	2019	Finland	1989–2014	50	50	Female (*n* = 50) Male (*n* = 0)	Angiosarcoma of the breast	OS, LRFS, DRFS	Tumor grade (G2 vs. G3); Radiotherapy (adjuvant); Chemotherapy (adjuvant); Age (continuous); Tumor size (continuous); Reconstructive surgery	univariate	N/A	Mean age 70.4 (SD 9.1)	5.4 (0.4–15.6)	50 Gy	7.7 (0.6–24.5)	7/9	extracted
Tsuda et al. [29]	2019	UK	1987–2017	47	25	Female (*n* = 15) Male (*n* = 10)	Bone sarcoma	LRFS	Resection strategy (radical (amputation) vs. limited (limb salvage))	univariate	N/A	42 (IQR 23–63)	40 months (IQR, 14–192)	N/A	16 (IQR 11–20)	7/9	calculated
Feinberg et al. [30]	2018	UK	1998–2015	38	36	Female (*n* = 36) Male (*n* = 0)	Angiosarcoma of the breast	OS, DSS	Treatment in multidisciplinary sarcoma center	univariate	N/A	69.5 (43–85)	center: 20.8 months (2.5–117) versus 17.8 months (5⋅8–117)	N/A	6 (3–21)	8/9	calculated
Italiano et al. [31]	2018	France	1990–2013	510	510	Female (*n* = 412) Male (*n* = 98)	Soft tissue sarcoma	OS, MFS, LRFS	Age (>66 years); Tumor size (>5.5 cm); Surgical margin (R0 vs. R1); Tumor grade (G1/G2 vs. G3); Histology	multivariate	age, sex, histologic subtype, grade, tumor size (median, 55 mm), tumor depth, tumor location, margin status, and presence of a lymphoedema	66.0 (15.0–93.0)	62.9 months (55.0–73.0)	N/A	N/A	7/9	extracted
Joo et al. [32]	2018	Korea	NA	43	42	Female (*n* = 29), Male (*n* = 13)	Soft tissue and bone sarcoma	OS, MFS, LRFS	Age (≥60 years); Tumor size (≥5 cm); Resection strategy (radical (wide margin) vs. limited (no wide margin)))	univariate	N/A	58.5 (13–80)	25.5 months (1–167)	49.7 Gy (12–72)	192 months (46–503)	5/9	calculated
Cohen-Hallaleh et al. [33]	2017	UK	2000–2014	49	49	Female (*n* = 49) Male (*n* = 0)	Angiosarcoma of the breast	OS, DMFS	Age (≥70 years); Tumor size (≥5 cm); Surgical margin (positive vs. negative); Chemotherapy (mixed cohort), Surgery (yes vs. no)	univariate/multivariate	N/A for OS, for DMFS: tumor size, margin status	72 (51–93)	N/A	50 Gy (40–54)	7.5 (1–26)	7/9	extracted and calculated
Li et al. [34]	2017	USA	1993–2015	76	76	Female (*n* = 76) Male (*n* = 0)	Angiosarcoma of the breast	DSS	Age (≥70 years); Surgical margin (negative vs. positive); Resection strategy (conservative vs. radical resection)	multivariate	age, radical resection, margin	conservative Group: 65 (57–69); radical Group: 60 (49–67)	27 months (IQR 11–60)	median: 60.9 Gy (conservative Group) 60.4 Gy (radical Group)	78 months (61–102), (conservative group) 88 months (76–138), (radical group)	9/9	extracted
Yin et al. [35]	2017	USA	1973–2012	472	173	Female (*n* = 173) Male (*n* = 0)	Angiosarcoma of the breast	OS	Tumor grade (G1 vs. G2 vs. G3); Radiotherapy (adjuvant); Age (≥ 60 years); Resection strategy (radical (Mastectomy) vs. limited (breast conservative surgery))	univariate/multivariate (Radiation)	influence of patient, pathological and treatment-related characteristics on death risk, adjusted by age, race, tumor stage, tumor grade, number of primaries	70–74	N/A	N/A	N/A	9/9	extracted
Linthorst et al. [36]	2013	Germany	2000–2011	24	23	N/A potential: Female (*n* = 24) Male (*n* = 0)	Angiosarcoma of the chest wall	OS	Surgery (yes vs. no)	univariate	N/A	70 (46–88)	12 months (1–78)	50 Gy (40–52)	106 months (45–212)	5/9	calculated
Torres et al. [37]	2013	USA	1993–2011	95	95	Female (*n* = 95) Male (*n* = 0)	Angiosarcoma of the breast	DSS	Age (>55 years); Surgical margin (negative vs. positive); Tumor size (>10 cm); Chemotherapy (mixed cohort)	univariate	N/A	71 (34–92)	10.3 (2.4–31.8)	60 Gy (46–65)	7 (1.4–26)	7/9	extracted
D’Angelo et al. [38]	2013	USA	1982–2011	79	79	Female (*n* = 79) Male (*n* = 0)	Angiosarcoma of the breast	DSS	Age (>68 years); Tumor size (>5 cm, ≤10 cm vs. ≤5 cm & >10 cm vs. ≤5 cm)	multivariate	age, depth, size	68 (36–87)	4.5	N/A	7 (3–19)	7/9	extracted
Gladdy et al. [39]	2010	USA	1982–2007	130	130	Female (*n* = 75) Male (*n* = 55)	Soft tissue sarcoma	DSS	Tumor size (>5 cm); Surgical margin (Negative vs. Micro/gross positive)	multivariate	age, histologic type, tumor size, site, depth, margin status	58.5 (18–86)	26.7 months (0.4–263)	54 Gy (20–160 Gy)	10 (1.3–74)	9/9	extracted
Patel et al. [6]	2010	USA	1975–2007	16	16	Female (*n* = 7) Male (*n* = 9)	Bone sarcoma (skull)	OS	Resection strategy (radical (gross-total resection vs. limited subtotal resection))	univariate	N/A	35.19 (95% CI, 26.34–44.03)	N/A	53.42 Gy (95% CI 45.97–60.88)	12.54 (95% CI 9.42–15.67)	4/9	calculated
Neuhaus et al. [40]	2008	UK	1990–2005	67	67	N/A	Soft tissue and bone sarcomas	DSS	Surgical margin (positive vs. negative)	univariate	N/A	58 (31–88)	53 months (5–141)	50 Gy (30–110)	11 (3–36)	7/9	extracted
Cha et al. [41]	2004	USA	1982–2001	123	111	Female (*n* = 66) Male (*n* = 57)	Soft tissue sarcoma	OS	Tumor grade (low vs. high); Surgical margin (negative vs. micro positive vs. gross positive)	univariate	N/A	62 (22–88)	12 (0.5–57)	N/A	101 (6–534) months	7/9	calculated

Abbreviations: overall survival (OS), disease specific survival (DSS), local recurrence free survival (LRFS), distant recurrence free survival (DRFS), metastasis free survival (MFS), relapse free survival (RFS), 95% confidence interval (95% CI), interquartile range (IQR), standard deviation (SD), gray (Gy), not applicable (N/A).

**Table 2:** Pooled hazard ratios and sensitivity analysis of prognostic factors in post-radiation sarcoma.

Risk Factor	OS	Random/ Fixed	I^2^	Sensitivity Analysis	DSS	Random/ Fixed	I^2^	Sensitivity Analysis	LRFS	Random/ Fixed	I^2^	Sensitivity Analysis	DRFS	Random/ Fixed	I^2^	sensitivity Analysis	Effect
Age	1.85 [1.28, 2.69]	Random	58%	1.87 [1.23, 2.82]	1.16 [1.07, 1.27]	Fixed	46%	1.68 [0.64, 4.36]	1.03 [1.00, 1.06]	Fixed	0%	1.02 [0.99, 1.06]	1.00 [0.94, 1.08]	Random	68%	1.53 [0.83, 2.82]	HR > 1 favors younger age
Tumor size	1.54 [1.02, 2.35]	Random	73%	1.24 [1.10, 1.41]	2.06 [1.34, 3.18]	Fixed	24%	2.42 [1.39, 4.19]	1.00 [0.93, 1.08]	Fixed	0%	0.99 [0.89, 1.11]	1.06 [0.97, 1.15]	Fixed	0%	1.04 [0.92, 1.17]	HR > 1 favors smaller Size
Grading	2.38 [1.86, 3.04]	Fixed	0%	2.74 [1.84, 4.06]	N/A	N/A	N/A	N/A	N/A	N/A	N/A	N/A	2.60 [1.65, 4.09]	Fixed	3%	2.60 [1.65, 4.09]	HR > 1 favors lower Grading
Multifocal disease versus single lesion	1.01 [0.70, 1.45]	Fixed	0%	1.00 [0.68, 1.46]	N/A	N/A	N/A	N/A	N/A	N/A	N/A	N/A	N/A	N/A	N/A	N/A	HR< 1 favors single lesion
Deep vs. superficial tumor location	2.58 [1.70, 3.90]	Fixed	0%	2.58 [1.70, 3.90]	N/A	N/A	N/A	N/A	N/A	N/A	N/A	N/A	N/A	N/A	N/A	N/A	HR > 1 favors superficial localized tumor
Local expertise	N/A	N/A	N/A	N/A	0.27 [0.14–0.53]	Fixed	0%	0.31 [0.15, 0.66]	N/A	N/A	N/A	N/A	N/A	N/A	N/A	N/A	HR < 1 favors specialized sarcoma center
Positive Margin	2.22 [1.75, 2.80]	Fixed	27%	2.14 [1.58, 2.90]	2.13 [1.45, 3.13]	Fixed	16%	2.64 [1.58, 4.42]	4.19 [1.66, 10.62]	Fixed	1%	5.71 [1.89, 17.25]	1.94 [1.31, 2.85]	Fixed	31%	1.80 [1.20, 2.70]	HR > 1 favors negative margin
Resection strategy radical vs. limited	0.54 [0.23, 1.22]	Random	57%	1.26 [0.60, 2.66]	0.53 [0.3, 0.94]	Fixed	20%	0.16 [0.03, 0.80]	0.43 [0.19, 0.94]	Fixed	49%	0.43 [0.19, 0.94]	N/A	N/A	N/A	N/A	HR < 1 favors radical surgery
Radiotherapy for PRS at any time	1.37 [0.94, 1.98]	Fixed	32%	1.77 [1.01, 3.11]	N/A	N/A	N/A	N/A	0.32 [0.04, 2.67]	Fixed	0%	N/A	N/A	N/A	N/A	N/A	HR < 1 favors Radiotherapy
Chemotherapy for PRS at any time	0.82 [0.60, 1.12]	Fixed	31%	0.92 [0.66, 1.28]	0.85 [0.33, 2.20]	Random	73%	0.67 [0.25, 1.81]	0.45 [0.31, 0.66]	Fixed	0%	0.43 [0.29, 0.63]	1.06 [0.59, 1.93]	Fixed	0%	1.06 [0.59, 1.93]	HR < 1 favors Chemotherapy
Surgery vs. No Surgery	4.37 [1.34, 14.25]	Random	88%	34.30 [11.92, 98.68]	N/A	N/A	N/A	N/A	N/A	N/A	N/A	N/A	N/A	N/A	N/A	N/A	HR > 1 favors surgery
Histology: Angiosarcoma	N/A	N/A	N/A	N/A	N/A	N/A	N/A	N/A	1.81 [0.64, 5.15]	Random	54%	1.30 [1.03, 1.64]	N/A	N/A	N/A	N/A	HR > 1 favors other than angiosarcoma

Abbreviations: overall survival (OS), disease specific survival (DSS), hazard ratio (HR), HR [95% confidence interval], post-radiation sarcoma (PRS), not applicable (N/A).

## Data Availability

All data relevant to the study are included in the article and its Supplementary Materials.

## Supplementary Materials

The following supporting information can be downloaded at: https://www.mdpi.com/article/10.3390/cancers18152414/s1, Supplementary Figure S1: Forest plots for age, including overall survival, disease-specific survival, local recurrence-free survival, distant recurrence-free survival, and continuous-variable analyses. Supplementary Figure S2: Forest plots for tumor size, including overall survival, disease-specific survival, local recurrence-free survival, distant recurrence-free survival, and continuous-variable analyses. Supplementary Figure S3: Forest plots for histopathology, including tumor grading and angiosarcoma versus non-angiosarcoma comparisons. Supplementary Figure S4: Forest plots for multifocality and tumor depth. Supplementary Figure S5: Forest plot for treatment at a multidisciplinary sarcoma center versus no multidisciplinary sarcoma center. Supplementary Figure S6: Forest plots for surgical treatment strategies, including radical versus limited resection, surgery versus no surgery, and reconstructive surgery. Supplementary Figure S7: Forest plots for radiotherapy at any time. Supplementary Figure S8: Forest plots for chemotherapy at any time. Supplementary Table S1: Quality assessment of included studies using the Newcastle–Ottawa Scale for cohort studies. Supplementary Table S2: Distribution of tumor grade across included studies and grading systems used. Supplementary Table S3: Overview of systemic treatment regimens and agents used across included studies. Supplementary Table S4: Full electronic search strategy. Supplementary Table S5: PRISMA 2020 checklist. Supplementary Table S6: Subgroup analysis of prognostic factors in post-radiation sarcoma. Supplementary Text: Narrative overview of sex, post-radiation sarcoma localization, reconstructive surgery, and histopathology.

## Abbreviations

The following abbreviations are used in this manuscript:

AJCC 7	American Joint Committee on Cancer, 7th edition
CanSaRCC	Canadian Sarcoma Research and Clinical Collaboration
CI	Confidence interval
CNS	Central nervous system
CT	Chemotherapy
DMFS	Distant metastasis-free survival
DRFS	Distant recurrence-free survival
DSS	Disease-specific survival
FNCLCC	Fédération nationale des centres de lutte contre le cancer
G1	Grade 1
G2	Grade 2
G3	Grade 3
Gy	Gray
HR	Hazard ratio
I^2^	I-squared statistic for heterogeneity
IQR	Interquartile range
LRFS	Local recurrence-free survival
MFS	Metastasis-free survival
MFH	Malignant fibrous histiocytoma
MPNST	Malignant peripheral nerve sheath tumor
N/A	Not applicable
NOS	Newcastle–Ottawa Scale
OS	Overall survival
PLD	Pegylated liposomal doxorubicin
PRISMA	Preferred Reporting Items for Systematic Reviews and Meta-Analyses
PROSPERO	International Prospective Register of Systematic Reviews
PRS	Post-radiation sarcoma
R0	Microscopically margin-negative resection
R1	Microscopically margin-positive resection
R2	Macroscopically incomplete resection/macroscopic residual disease
RFS	Relapse-free survival
RIS	Radiation-induced sarcoma
RT	Radiotherapy
SD	Standard deviation
UPS	Undifferentiated pleomorphic sarcoma
