# Supplementary material for: Prognostic Factors and Outcomes for Post-Radiation Sarcoma—A Systematic Review and Meta-Analysis
Source: Cancers (Basel). 2026 Jul 27;18(15):2414. doi: 10.3390/cancers18152414 (PMC13465409; doi:10.3390/cancers18152414)
Supplement: Supplementary file 1 [file cancers-18-02414-s001.zip › cancers-4452077-supplementary.pdf]

Supplement: Risk Factors and Outcomes for Post-Radiation Sarcoma—A Systematic Review and Meta-Analysis

Figure S1 Forest Plot: Age

Figure S1a Overall Survival

Study or Subgroup	log[HR]	SE	Weight	Hazard ratio IV, Random, 95% CI
**1.1.1 Age ≥ 60**				
Italiano et al.[a]	0.336472	0.162686	20.9%	1.40 [1.02 , 1.93]
Joo et al[b]	-1.07881	0.577955	7.5%	0.34 [0.11 , 1.06]
Kao et al.[c]	0.883768	0.387929	12.2%	2.42 [1.13 , 5.18]
Ribeiro et al.[d]	0.854415	0.412997	11.4%	2.35 [1.05 , 5.28]
Yin et al.[e]	0.647103	0.296429	15.4%	1.91 [1.07 , 3.41]
**Subtotal (Wald[f])**			**67.5%**	**1.54 [0.97 , 2.45]**
Test for overall effect: Z = 1.84 (P = 0.07)				
Heterogeneity: Tau² (DL[g]) = 0.16; Chi² = 10.11, df = 4 (P = 0.04); I² = 60%				
**1.1.2 Age ≥70**				
Cohen-Hallaleh et al.[e]	0.662688	0.472621	9.8%	1.94 [0.77 , 4.90]
Michot et al.[h]	0.887891	0.244411	17.6%	2.43 [1.51 , 3.92]
Wong et al.[i]	1.964171	0.747912	5.1%	7.13 [1.65 , 30.88]
**Subtotal (Wald[f])**			**32.5%**	**2.57 [1.60 , 4.11]**
Test for overall effect: Z = 3.92 (P < 0.0001)				
Heterogeneity: Tau² (DL[g]) = 0.03; Chi² = 2.26, df = 2 (P = 0.32); I² = 12%				
**Total (Wald[f])**			**100.0%**	**1.85 [1.28 , 2.69]**

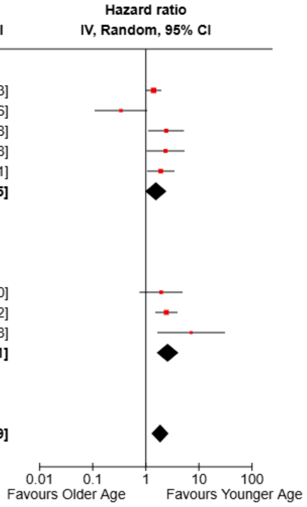

Test for overall effect: Z = 3.24 (P = 0.001)
Test for subgroup differences: Chi² = 2.27, df = 1 (P = 0.13), I² = 55.9%
Heterogeneity: Tau² (DL[g]) = 0.15; Chi² = 16.59, df = 7 (P = 0.02); I² = 58%

Footnotes
[a]Age > 66; Multivariate
[b]Univariate, calculated
[c]Age >62; Univariate
[d]Multivariate
[e]Univariate
[f]CI calculated by Wald-type method.
[g]Tau² calculated by DerSimonian and Laird method.
[h]Age ≥73; Multivariate
[i]Age > 72; Univariate

Figure S1b Disease Specific Survival

Study or Subgroup	log[HR]	SE	Weight	Hazard ratio IV, Fixed, 95% CI
**1.2.1 Age >40**				
Lazarides et al.[a]	0.955511	0.62673	0.5%	2.60 [0.76 , 8.88]
**Subtotal**			**0.5%**	**2.60 [0.76 , 8.88]**
Test for overall effect: Z = 1.52 (P = 0.13)				
Heterogeneity: Not applicable				
**1.2.2 Age >55**				
Torres et al.[a]	0.559616	0.350967	1.6%	1.75 [0.88 , 3.48]
**Subtotal**			**1.6%**	**1.75 [0.88 , 3.48]**
Test for overall effect: Z = 1.59 (P = 0.11)				
Heterogeneity: Not applicable				
**1.2.3 Age ≥65**				
D'Angelo et al.[b]	1.134623	0.486506	0.8%	3.11 [1.20 , 8.07]
Garayua-Cruz et al.[c]	0.425268	0.877553	0.3%	1.53 [0.27 , 8.54]
**Subtotal**			**1.1%**	**2.63 [1.14 , 6.06]**
Test for overall effect: Z = 2.27 (P = 0.02)				
Heterogeneity: Chi² = 0.50, df = 1 (P = 0.48); I² = 0%				
**1.2.4 Age ≥70**				
Li et al.[d]	0.131028	0.044871	96.8%	1.14 [1.04 , 1.24]
**Subtotal**			**96.8%**	**1.14 [1.04 , 1.24]**
Test for overall effect: Z = 2.92 (P = 0.003)				
Heterogeneity: Not applicable				
**Total**			**100.0%**	**1.16 [1.07 , 1.27]**

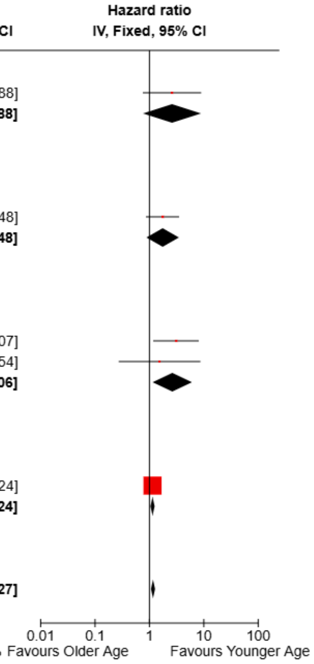

Test for overall effect: Z = 3.42 (P = 0.0006)
Test for subgroup differences: Chi² = 6.89, df = 3 (P = 0.08), I² = 56.4%
Heterogeneity: Chi² = 7.39, df = 4 (P = 0.12); I² = 46%

Footnotes
[a]Univariate
[b]Age >68; Multivariate
[c]Age ≥65; Univariate
[d]Multivariate

Figure S1c Local Recurrence Free Survival Based on Continuous Variable (Angiosarcoma Only)

Study or Subgroup	log[HR]	SE	Weight	Hazard ratio IV, Fixed, 95% CI
Kuba et al.[a]	0.023717	0.016191	77.1%	1.02 [0.99 , 1.06]
Salminen et al.[b]	0.044973	0.029731	22.9%	1.05 [0.99 , 1.11]
**Total**			**100.0%**	**1.03 [1.00 , 1.06]**

Test for overall effect: Z = 2.01 (P = 0.04)

Heterogeneity: Chi² = 0.39, df = 1 (P = 0.53); I² = 0%

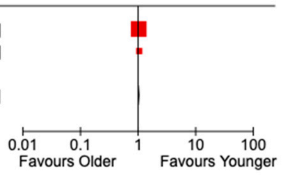

Footnotes
[a]Multivariate
[b]Univariate

Figure S1d Distant Recurrence Free Survival Based on Continuous Variable (Angiosarcoma Only)

Study or Subgroup	log[HR]	SE	Weight	Hazard ratio IV, Random, 95% CI
Kuba et al.[a]	-0.026344	0.021217	57.4%	0.97 [0.93 , 1.02]
Salminen et al.[b]	0.046884	0.035268	42.6%	1.05 [0.98 , 1.12]
**Total (Wald[c])**			**100.0%**	**1.00 [0.94 , 1.08]**

Test for overall effect: Z = 0.13 (P = 0.89)

Heterogeneity: Tau² (DL[d]) = 0.00; Chi² = 3.17, df = 1 (P = 0.08); I² = 68%

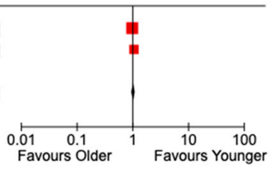

Footnotes
[a]Multivariate
[b]Univariate
[c]CI calculated by Wald-type method.
[d]Tau² calculated by DerSimonian and Laird method.

Figure S1e Overall Survival Based on Continuous Variable

Study or Subgroup	log[HR]	SE	Weight	Hazard ratio IV, Random, 95% CI
Kuba et al.[a]	-0.014099	0.018886	37.7%	0.99 [0.95 , 1.02]
Palassini et al.[b]	0.058269	0.021776	35.0%	1.06 [1.02 , 1.11]
Salminen et al.[b]	0.026642	0.030779	27.2%	1.03 [0.97 , 1.09]
**Total (Wald[c])**			**100.0%**	**1.02 [0.98 , 1.07]**

Test for overall effect: Z = 0.93 (P = 0.35)

Heterogeneity: Tau² (DL[d]) = 0.00; Chi² = 6.38, df = 2 (P = 0.04); I² = 69%

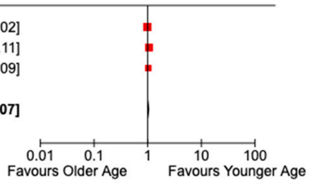

Footnotes
[a]Multivariate
[b]Univariate
[c]CI calculated by Wald-type method.
[d]Tau² calculated by DerSimonian and Laird method.

Figure S1f Distant Recurrence Free Survival

Study or Subgroup	log[HR]	SE	Weight	Hazard ratio IV, Random, 95% CI
Joo et al[a]	-0.967584	0.534013	45.2%	0.38 [0.13 , 1.08]
Michot et al.[b]	0.425268	0.312239	54.8%	1.53 [0.83 , 2.82]
**Total (Wald[c])**			**100.0%**	**0.82 [0.21 , 3.17]**

Test for overall effect: Z = 0.29 (P = 0.77)

Heterogeneity: Tau² (DL[d]) = 0.78; Chi² = 5.07, df = 1 (P = 0.02); I² = 80%

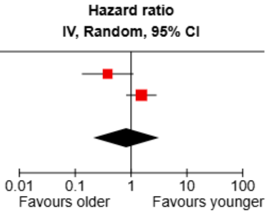

Footnotes
[a]Age ≥66 , Univariate, calculated
[b]Age ≥73, univariate
[c]CI calculated by Wald-type method.
[d]Tau² calculated by DerSimonian and Laird method.

Figure S2. Forest Plot: Tumor size

Figure S2a Overall Survival

Study or Subgroup	log[HR]	SE	Weight	Hazard ratio IV, Random, 95% CI
**2.1.1 Size ≥ 4.5 cm**				
Michot et al.[a]	0.722706	0.258813	22.2%	2.06 [1.24 , 3.42]
**Subtotal**			**22.2%**	**2.06 [1.24 , 3.42]**
Test for overall effect: Z = 2.79 (P = 0.005)				
Heterogeneity: Not applicable				
**2.1.2 Size ≥ 5-5.5cm**				
Cohen-Hallaleh et al.[b]	1.644805	0.663494	7.9%	5.18 [1.41 , 19.02]
Italiano et al.[a]	0.470004	0.176827	26.9%	1.60 [1.13 , 2.26]
Wong et al.[b]	-0.994793	0.781566	6.1%	0.37 [0.08 , 1.71]
**Subtotal (Wald[c])**			**40.9%**	**1.56 [0.52 , 4.67]**
Test for overall effect: Z = 0.79 (P = 0.43)				
Heterogeneity: Tau² (DL[d]) = 0.65; Chi² = 6.65, df = 2 (P = 0.04); I² = 70%				
**2.1.3 Size ≥ 6.5 cm**				
Kao et al.[a]	1.172482	0.973456	4.2%	3.23 [0.48 , 21.77]
**Subtotal**			**4.2%**	**3.23 [0.48 , 21.77]**
Test for overall effect: Z = 1.20 (P = 0.23)				
Heterogeneity: Not applicable				
**2.1.4 Largest tumor diameter (4.7-7.2 cm)**				
Ribeiro et al.[a]	0.086178	0.035325	32.7%	1.09 [1.02 , 1.17]
**Subtotal**			**32.7%**	**1.09 [1.02 , 1.17]**
Test for overall effect: Z = 2.44 (P = 0.01)				
Heterogeneity: Not applicable				
**Total (Wald[c])**			**100.0%**	**1.54 [1.02 , 2.35]**

Test for overall effect: Z = 2.03 (P = 0.04)
Test for subgroup differences: Chi² = 7.53, df = 3 (P = 0.06), I² = 60.2%
Heterogeneity: Tau² (DL[d]) = 0.14; Chi² = 18.72, df = 5 (P = 0.002); I² = 73%

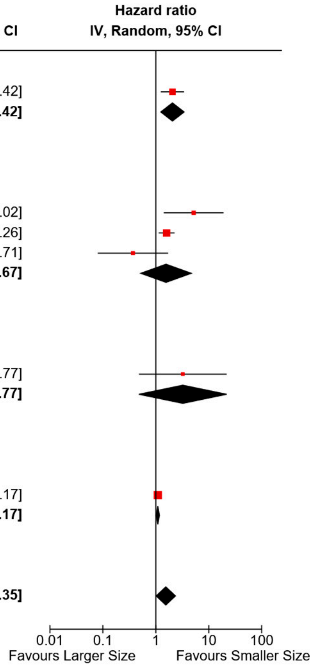

Footnotes
[a]Multivariate
[b]Univariate
[c]CI calculated by Wald-type method.
[d]Tau² calculated by DerSimonian and Laird method.

Figure S2b Disease Specific Survival

Study or Subgroup	log[HR]	SE	Weight	Hazard ratio IV, Fixed, 95% CI
**2.2.1 Size ≥ 5-5.5cm**				
D'Angelo et al.[a]	0.231112	0.49259	20.1%	1.26 [0.48 , 3.31]
D'Angelo et al.[b]	0.683097	0.694155	10.1%	1.98 [0.51 , 7.72]
Gladdy et al.[c]	1.360977	0.392978	31.6%	3.90 [1.81 , 8.42]
**Subtotal**			**61.8%**	**2.42 [1.39 , 4.19]**
Test for overall effect: Z = 3.14 (P = 0.002)				
Heterogeneity: Chi² = 3.31, df = 2 (P = 0.19); I² = 40%				
**2.2.2 Size ≥ 10-11 cm**				
Lazarides et al.[d]	0.788457	0.469813	22.1%	2.20 [0.88 , 5.52]
Torres et al.[e]	0.029559	0.549504	16.1%	1.03 [0.35 , 3.02]
**Subtotal**			**38.2%**	**1.60 [0.79 , 3.22]**
Test for overall effect: Z = 1.31 (P = 0.19)				
Heterogeneity: Chi² = 1.10, df = 1 (P = 0.29); I² = 9%				
**Total**			**100.0%**	**2.06 [1.34 , 3.18]**

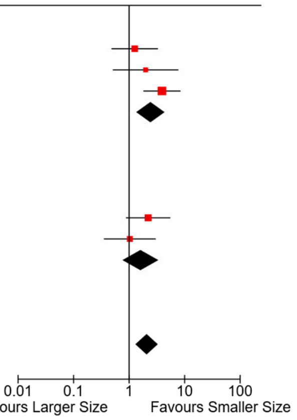

Test for overall effect: Z = 3.28 (P = 0.001)
Test for subgroup differences: Chi² = 0.83, df = 1 (P = 0.36), I² = 0%
Heterogeneity: Chi² = 5.25, df = 4 (P = 0.26); I² = 24%

Footnotes
[a]Multivariate; >5cm,⩽10cm vs⩽5cm
[b]Multivariate; >10cm vs⩽5cm
[c]Multivariate
[d]11cm; Univariate
[e]10cm, Univariate

Figure S2c Overall Survival Based on Continuous Variable (Angiosarcoma Only)

Study or Subgroup	log[HR]	SE	Weight	Hazard ratio IV, Fixed, 95% CI
Kuba et al.[a]	0.218332	0.064629	33.5%	1.24 [1.10 , 1.41]
Palassini et al.[b]	0.086178	0.06529	32.8%	1.09 [0.96 , 1.24]
Salminen et al.[b]	0.052592	0.064447	33.7%	1.05 [0.93 , 1.20]
**Total**			**100.0%**	**1.13 [1.05 , 1.21]**

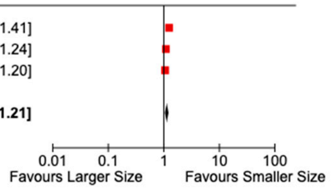

Test for overall effect: Z = 3.18 (P = 0.001)

Heterogeneity: Chi² = 3.68, df = 2 (P = 0.16); I² = 46%

Footnotes
[a]Multivariate
[b]Univariate

Figure S2d Local Recurrence Free Survival Based on Continuous Variable (Angiosarcoma Only)

Study or Subgroup	log[HR]	SE	Weight	Hazard ratio IV, Fixed, 95% CI
Kuba et al.[a]	-0.007025	0.054627	52.4%	0.99 [0.89 , 1.11]
Salminen et al.[b]	0.013903	0.057263	47.6%	1.01 [0.91 , 1.13]
**Total**			**100.0%**	**1.00 [0.93 , 1.08]**

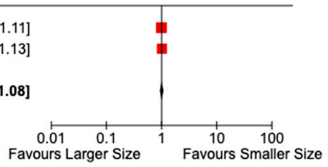

Test for overall effect: Z = 0.07 (P = 0.94)

Heterogeneity: Chi² = 0.07, df = 1 (P = 0.79); I² = 0%

Footnotes
[a]Multivariate
[b]Univariate

Figure S2e Distant Recurrence Free Survival Based on Continuous Variable (Angiosarcoma Only)

Study or Subgroup	log[HR]	SE	Weight	Hazard ratio IV, Fixed, 95% CI
Kuba et al.[a]	0.035367	0.062871	50.1%	1.04 [0.92 , 1.17]
Salminen et al.[b]	0.072321	0.062976	49.9%	1.08 [0.95 , 1.22]
**Total**			**100.0%**	**1.06 [0.97 , 1.15]**

Test for overall effect: Z = 1.21 (P = 0.23)

Heterogeneity: Chi² = 0.17, df = 1 (P = 0.68); I² = 0%

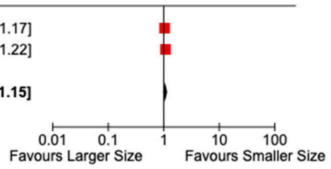

Footnotes
[a]Multivariate
[b]Univariate

Figure S3. Forest Plot: Histopathology

Figure S3a G1 versus G2 versus G3: Overall Survival

Study or Subgroup	log[HR]	SE	Weight	Hazard ratio IV, Fixed, 95% CI
**3.1.1 G1 vs. G2**				
Mito et al.[a]	-0.18633	0.717465	3.0%	0.83 [0.20 , 3.39]
Ribeiro et al.[a]	1.405116	0.685655	3.3%	4.08 [1.06 , 15.63]
Spalek et al.[b]	0.482426	1.06511	1.4%	1.62 [0.20 , 13.07]
Yin et al.[b]	0.582216	0.519452	5.8%	1.79 [0.65 , 4.95]
**Subtotal**			**13.5%**	**1.83 [0.94 , 3.55]**
Test for overall effect: Z = 1.77 (P = 0.08)				
Heterogeneity: Chi² = 2.59, df = 3 (P = 0.46); I² = 0%				
**3.1.2 G1 vs. G3**				
Ribeiro et al.[a]	1.938742	0.682773	3.4%	6.95 [1.82 , 26.49]
Spalek et al.[b]	0.81978	1.028106	1.5%	2.27 [0.30 , 17.03]
Yin et al.[b]	0.770108	0.462888	7.3%	2.16 [0.87 , 5.35]
**Subtotal**			**12.1%**	**3.00 [1.49 , 6.07]**
Test for overall effect: Z = 3.06 (P = 0.002)				
Heterogeneity: Chi² = 2.09, df = 2 (P = 0.35); I² = 4%				
**3.1.3 G2 vs G3**				
Kao et al.[b]	0.920283	0.646363	3.7%	2.51 [0.71 , 8.91]
Mito et al.[a]	1.629241	0.401547	9.7%	5.10 [2.32 , 11.20]
Salminen et al.[b]	3.248939	2.206971	0.3%	25.76 [0.34 , 1947.93]
**Subtotal**			**13.8%**	**4.37 [2.26 , 8.46]**
Test for overall effect: Z = 4.37 (P < 0.0001)				
Heterogeneity: Chi² = 1.53, df = 2 (P = 0.47); I² = 0%				
**3.1.4 G1/G2 vs G3**				
Cha et al.[b]	1.022451	0.371571	11.3%	2.78 [1.34 , 5.76]
Italiano et al.[a]	0.641854	0.287879	18.9%	1.90 [1.08 , 3.34]
Michot et al.[b]	0.698135	0.226938	30.4%	2.01 [1.29 , 3.14]
**Subtotal**			**60.6%**	**2.10 [1.53 , 2.88]**
Test for overall effect: Z = 4.61 (P < 0.00001)				
Heterogeneity: Chi² = 0.73, df = 2 (P = 0.69); I² = 0%				
**Total**			**100.0%**	**2.38 [1.86 , 3.04]**

Test for overall effect: Z = 6.93 (P < 0.00001)
Test for subgroup differences: Chi² = 4.88, df = 3 (P = 0.18), I² = 38.6%
Heterogeneity: Chi² = 11.83, df = 12 (P = 0.46); I² = 0%

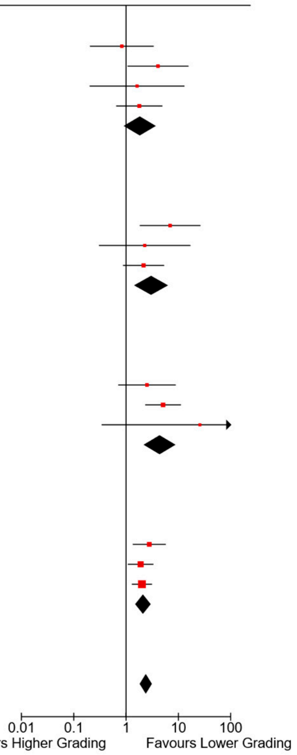

Footnotes
[a]Multivariate
[b]Univariate

Figure S3b G1/G2 versus G3: Distant Recurrence Free Survival Based on Univariate Hazard

Study or Subgroup	log[HR]	SE	Weight	Hazard ratio IV, Fixed, 95% CI
Italiano et al.[a]	1.223775	0.420584	30.2%	3.40 [1.49 , 7.75]
Michot et al.[b]	0.652325	0.316384	53.3%	1.92 [1.03 , 3.57]
Torres et al.[b]	1.442202	0.56816	16.5%	4.23 [1.39 , 12.88]
**Total**			**100.0%**	**2.60 [1.65 , 4.09]**

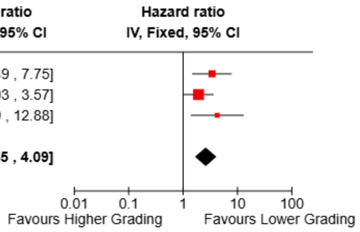

Test for overall effect: Z = 4.14 (P < 0.0001)

Heterogeneity: Chi² = 2.06, df = 2 (P = 0.36); I² = 3%

Footnotes

[a]Soft tissue sarcoma

[b]Angiosarcoma of the breast

Figure S3c Angiosarcoma versus no Angiosarcoma: Local Recurrence Free Survival

Study or Subgroup	log[HR]	SE	Weight	Hazard ratio IV, Random, 95% CI
Italiano et al.[a]	0.262364	0.11723	72.1%	1.30 [1.03 , 1.64]
Kuba et al.[b]	1.451614	0.799512	27.9%	4.27 [0.89 , 20.46]
**Total (Wald[c])**			**100.0%**	**1.81 [0.64 , 5.15]**

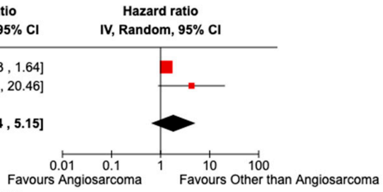

Test for overall effect: Z = 1.11 (P = 0.27)

Heterogeneity: Tau² (DL[d]) = 0.38; Chi² = 2.17, df = 1 (P = 0.14); I² = 54%

Footnotes

[a]Multivariate

[b]Univariate; calculated

[c]CI calculated by Wald-type method.

[d]Tau² calculated by DerSimonian and Laird method.

Figure S4. Forest Plot: Multifocality

Figure S4a Multifocal Disease versus Single Lesion: Overall Survival Based on Univariate Hazard Ratios (Angiosarcoma Only)

Study or Subgroup	log[HR]	SE	Weight	Hazard ratio IV, Fixed, 95% CI
Michot et al.	-0.030459	0.228766	65.8%	0.97 [0.62 , 1.52]
Palassini et al.	0.067659	0.367962	25.4%	1.07 [0.52 , 2.20]
Wong et al.	0.126633	0.629	8.7%	1.14 [0.33 , 3.89]
**Total**			**100.0%**	**1.01 [0.70 , 1.45]**

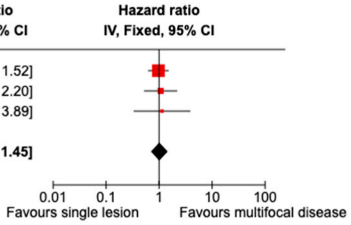

Test for overall effect: Z = 0.04 (P = 0.96)

Heterogeneity: Chi² = 0.09, df = 2 (P = 0.96); I² = 0%

Figure S4b Deep Tumors vs Superficial Tumors: Overall Survival Based on Multivariate Hazard Ratios

Study or Subgroup	log[HR]	SE	Weight	Hazard ratio IV, Fixed, 95% CI
Michot et al.[a]	0.875469	0.268251	62.3%	2.40 [1.42 , 4.06]
Mito et al.[b]	1.064711	0.345005	37.7%	2.90 [1.47 , 5.70]
**Total**			**100.0%**	**2.58 [1.70 , 3.90]**

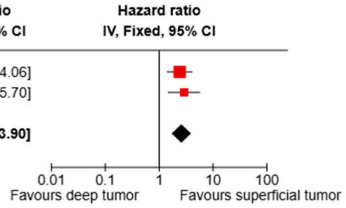

Test for overall effect: Z = 4.47 (P < 0.00001)

Heterogeneity: Chi² = 0.19, df = 1 (P = 0.66); I² = 0%

Footnotes

[a]deep vs superficial

[b]Superficial chest or extremity vs. Deep chest, abdomen, pelvis

Figure S5. Forest Plot: Sarcoma Multidisciplinary Sarcoma Center versus No Sarcoma Multidisciplinary Sarcoma Center: Disease Specific Survival: Univariate Hazard Ratios (Angiosarcoma Only)

Study or Subgroup	log[HR]	SE	Weight	Hazard ratio IV, Fixed, 95% CI
Feinberg et al.[a]	-1.771957	0.722772	22.2%	0.17 [0.04 , 0.70]
Guram et al.[b]	-1.171183	0.385583	77.8%	0.31 [0.15 , 0.66]
**Total**			**100.0%**	**0.27 [0.14 , 0.53]**

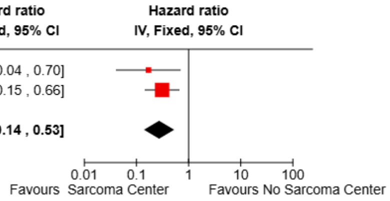

Test for overall effect: Z = 3.83 (P = 0.0001)

Heterogeneity: Chi² = 0.54, df = 1 (P = 0.46); I² = 0%

Footnotes

[a]calculated

[b]Multivariate

Figure S6. Forest Plot: Surgery

Figure S6a Radical versus limited resection: Overall Survival

Study or Subgroup	log[HR]	SE	Weight	Hazard ratio IV, Random, 95% CI
Joo et al[a]	-1.171183	0.587405	24.2%	0.31 [0.10 , 0.98]
Patel et al.[a]	-1.771957	0.884133	15.2%	0.17 [0.03 , 0.96]
Salminen et al.[b]	-0.543005	0.506876	27.5%	0.58 [0.22 , 1.57]
Yin et al.[c]	0.231112	0.381804	33.2%	1.26 [0.60 , 2.66]
**Total (Wald[d])**			**100.0%**	**0.54 [0.23 , 1.22]**

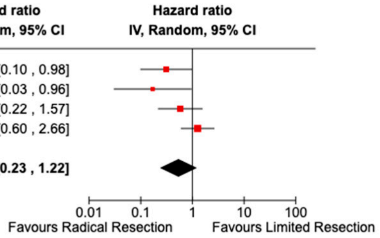

Test for overall effect: Z = 1.48 (P = 0.14)

Heterogeneity: Tau² (DL[e]) = 0.39; Chi² = 6.94, df = 3 (P = 0.07); I² = 57%

Footnotes

[a]Univariate; calculated

[b]Univariate

[c]Multivariate

[d]CI calculated by Wald-type method.

[e]Tau² calculated by DerSimonian and Laird method.

Figure S6b Radical versus limited resection: Disease Specific Survival

Study or Subgroup	log[HR]	SE	Weight	Hazard ratio IV, Fixed, 95% CI
**6.2.1 Univariate**				
Guram et al.	-0.527633	0.398981	54.4%	0.59 [0.27 , 1.29]
Lazarides et al.[a]	-0.356675	0.514695	32.7%	0.70 [0.26 , 1.92]
**Subtotal**			**87.1%**	**0.63 [0.34 , 1.17]**
Test for overall effect: Z = 1.47 (P = 0.14)				
Heterogeneity: Chi² = 0.07, df = 1 (P = 0.79); I² = 0%				
**6.2.2 Multivariate**				
Li et al.	-1.832581	0.821157	12.9%	0.16 [0.03 , 0.80]
**Subtotal**			**12.9%**	**0.16 [0.03 , 0.80]**
Test for overall effect: Z = 2.23 (P = 0.03)				
Heterogeneity: Not applicable				
**Total**			**100.0%**	**0.53 [0.30 , 0.94]**

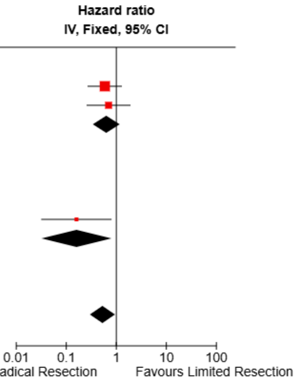

Test for overall effect: Z = 2.17 (P = 0.03)
Test for subgroup differences: Chi² = 2.42, df = 1 (P = 0.12), I² = 58.7%
Heterogeneity: Chi² = 2.49, df = 2 (P = 0.29); I² = 20%

Footnotes
[a]Amputation vs Limb Salvage

Figure S6c Radical versus Limited Resection: Local Recurrence Free Survival Based on Univariate Hazard

Study or Subgroup	log[HR]	SE	Weight	Hazard ratio IV, Fixed, 95% CI
Salminen et al.[a]	-0.525939	0.466425	75.1%	0.59 [0.24 , 1.47]
Tsuda et al.[b]	-1.832581	0.810743	24.9%	0.16 [0.03 , 0.78]
**Total**			**100.0%**	**0.43 [0.19 , 0.94]**

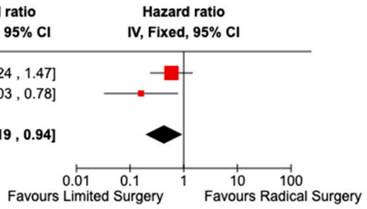

Test for overall effect: Z = 2.10 (P = 0.04)
Heterogeneity: Chi² = 1.95, df = 1 (P = 0.16); I² = 49%

Footnotes
[a]Radical Surgery Angiosarcoma
[b]limb salvage group and an amputation group in Osteosarcoma

Figure S6d Surgery versus no surgery: Overall Survival

Study or Subgroup	log[HR]	SE	Weight	Hazard ratio IV, Random, 95% CI
**6.4.1 univariate**				
Callesen et al.	0.993252	0.324742	26.6%	2.70 [1.43 , 5.10]
Cohen-Hallaleh et al.[a]	1.355835	0.411755	25.4%	3.88 [1.73 , 8.70]
Linthorst et al.[a]	0.165514	0.459734	24.7%	1.18 [0.48 , 2.91]
**Subtotal (Wald[b])**			**76.6%**	**2.40 [1.29 , 4.47]**
Test for overall effect: Z = 2.77 (P = 0.006)				
Heterogeneity: Tau² (DL[c]) = 0.15; Chi² = 3.86, df = 2 (P = 0.15); I² = 48%				
**6.4.2 multivariate**				
Mito et al.	3.535145	0.539173	23.4%	34.30 [11.92 , 98.68]
**Subtotal**			**23.4%**	**34.30 [11.92 , 98.68]**
Test for overall effect: Z = 6.56 (P < 0.00001)				
Heterogeneity: Not applicable				
**Total (Wald[b])**			**100.0%**	**4.37 [1.34 , 14.25]**

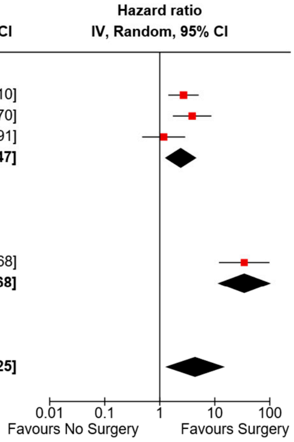

Test for overall effect: Z = 2.45 (P = 0.01)
Test for subgroup differences: Chi² = 18.08, df = 1 (P < 0.0001), I² = 94.5%
Heterogeneity: Tau² (DL[c]) = 1.26; Chi² = 24.18, df = 3 (P < 0.0001); I² = 88%

Footnotes
[a]calculated
[b]CI calculated by Wald-type method.
[c]Tau² calculated by DerSimonian and Laird method.

Figure S6e Reconstructive Surgery versus No Reconstructive Surgery: Overall Survival Based on Univariate Hazard Ratios

Study or Subgroup	log[HR]	SE	Weight	Hazard ratio IV, Fixed, 95% CI
Lazarides et al.	-0.693147	0.733785	32.5%	0.50 [0.12 , 2.11]
Salminen et al.	-0.543005	0.5095	67.5%	0.58 [0.21 , 1.58]
**Total**			**100.0%**	**0.55 [0.24 , 1.26]**

Test for overall effect: Z = 1.41 (P = 0.16)

Heterogeneity: Chi² = 0.03, df = 1 (P = 0.87); I² = 0%

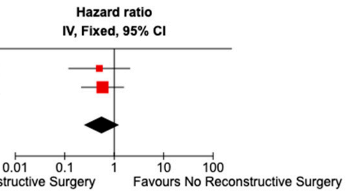

Figure S7. Forest Plot: Radiotherapy at any time

Figure S7a Overall Survival

Study or Subgroup	log[HR]	SE	Weight	Hazard ratio IV, Fixed, 95% CI
**7.1.1 Adjuvant Radiotherapy**				
Salminen et al.[a]	1.654794	1.060922	3.2%	5.23 [0.65 , 41.85]
Yin et al.[b]	0.57098	0.28773	43.3%	1.77 [1.01 , 3.11]
**Subtotal**			**46.5%**	**1.91 [1.11 , 3.29]**
Test for overall effect: Z = 2.32 (P = 0.02)				
Heterogeneity: Chi² = 0.97, df = 1 (P = 0.32); I² = 0%				
**7.1.2 Mixed (neoadjuvant/adjuvant/unspecified)**				
Kao et al.[a]	0.215111	0.376027	25.4%	1.24 [0.59 , 2.59]
Palassini et al.[a]	-0.673345	0.533998	12.6%	0.51 [0.18 , 1.45]
Spalek et al.[a]	0.270027	0.480949	15.5%	1.31 [0.51 , 3.36]
**Subtotal**			**53.5%**	**1.02 [0.62 , 1.70]**
Test for overall effect: Z = 0.08 (P = 0.93)				
Heterogeneity: Chi² = 2.23, df = 2 (P = 0.33); I² = 10%				
**Total**			**100.0%**	**1.37 [0.94 , 1.98]**

Test for overall effect: Z = 1.65 (P = 0.10)

Test for subgroup differences: Chi² = 2.69, df = 1 (P = 0.10), I² = 62.9%

Heterogeneity: Chi² = 5.89, df = 4 (P = 0.21); I² = 32%

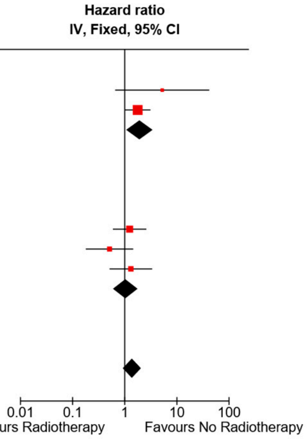

Footnotes

[a]Univariate

[b]Multivariate

Figure S7b Local Recurrence Free Survival

Study or Subgroup	log[HR]	SE	Weight	Hazard ratio IV, Fixed, 95% CI
**7.2.1 Radiotherapy at any time (Univariate)**				
Garayua-Cruz et al.[a]	-0.941609	1.132599	90.9%	0.39 [0.04 , 3.59]
Salminen et al.[b]	-3.036554	3.572668	9.1%	0.05 [0.00 , 52.76]
**Subtotal**			**100.0%**	**0.32 [0.04 , 2.67]**
Test for overall effect: Z = 1.05 (P = 0.29)				
Heterogeneity: Chi² = 0.31, df = 1 (P = 0.58); I² = 0%				
**Total**			**100.0%**	**0.32 [0.04 , 2.67]**

Test for overall effect: Z = 1.05 (P = 0.29)

Test for subgroup differences: Not applicable

Heterogeneity: Chi² = 0.31, df = 1 (P = 0.58); I² = 0%

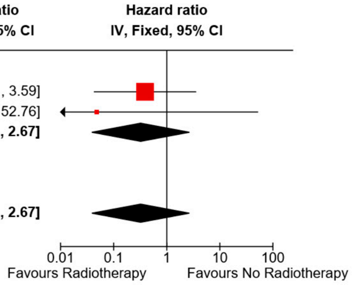

Footnotes

[a]Mixed Radiotherapy-Regimes

[b]postop Radiotherapy, CAVE: <1 favors Radiotherapy (lower CI was 0)

Figure S8. Forest Plot: Chemotherapy at any time

Figure S8a Overall Survival: Univariate Hazard Ratios

Study or Subgroup	log[HR]	SE	Weight	Hazard ratio IV, Fixed, 95% CI
**8.1.1 Adjuvant Chemotherapy**				
Salminen et al.	0.774727	0.652804	5.9%	2.17 [0.60 , 7.80]
**Subtotal**			**5.9%**	**2.17 [0.60 , 7.80]**
Test for overall effect: Z = 1.19 (P = 0.24)				
Heterogeneity: Not applicable				
**8.1.2 Mixed (neoadjuvant/adjuvant/unspecified)**				
Cohen-Hallaleh et al.	0.425268	0.753817	4.4%	1.53 [0.35 , 6.70]
Kao et al.[a]	0.307485	0.394707	16.1%	1.36 [0.63 , 2.95]
Palassini et al.	-0.510826	0.370666	18.3%	0.60 [0.29 , 1.24]
Spalek et al.	-0.150823	0.470488	11.3%	0.86 [0.34 , 2.16]
Wong et al.	-0.518858	0.632828	6.3%	0.60 [0.17 , 2.06]
**Subtotal**			**56.4%**	**0.88 [0.58 , 1.32]**
Test for overall effect: Z = 0.63 (P = 0.53)				
Heterogeneity: Chi² = 3.21, df = 4 (P = 0.52); I² = 0%				
**8.1.3 Neoadjuvant Chemotherapy**				
Michot et al.	-0.235722	0.278408	32.4%	0.79 [0.46 , 1.36]
van der Burg et al.[b]	-1.660731	0.683947	5.4%	0.19 [0.05 , 0.73]
**Subtotal**			**37.7%**	**0.65 [0.39 , 1.07]**
Test for overall effect: Z = 1.70 (P = 0.09)				
Heterogeneity: Chi² = 3.72, df = 1 (P = 0.05); I² = 73%				
**Total**			**100.0%**	**0.82 [0.60 , 1.12]**

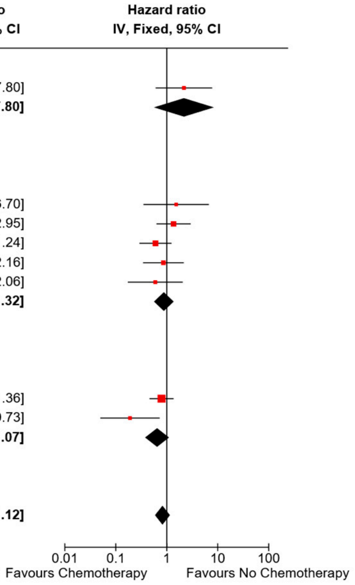

Test for overall effect: Z = 1.23 (P = 0.22)
Test for subgroup differences: Chi² = 3.19, df = 2 (P = 0.20), I² = 37.2%
Heterogeneity: Chi² = 10.12, df = 7 (P = 0.18); I² = 31%

Footnotes
[a]Authors didn't provide any Chemotherapy details
[b]calculated

Figure S8b Disease Specific Survival: Univariate Hazard Ratios

Study or Subgroup	log[HR]	SE	Weight	Hazard ratio IV, Random, 95% CI
**8.2.1 Neoadjuvant Chemotherapy**				
Guram et al.	-0.891598	0.399604	28.8%	0.41 [0.19 , 0.90]
**Subtotal**			**28.8%**	**0.41 [0.19 , 0.90]**
Test for overall effect: Z = 2.23 (P = 0.03)				
Heterogeneity: Not applicable				
**8.2.2 Mixed (neoadjuvant/adjuvant)**				
Garayua-Cruz et al.	1.121678	0.916806	15.7%	3.07 [0.51 , 18.52]
Lazarides et al.	-0.916291	0.428715	28.0%	0.40 [0.17 , 0.93]
Torres et al.	0.652325	0.445232	27.5%	1.92 [0.80 , 4.59]
**Subtotal (Wald[a])**			**71.2%**	**1.18 [0.33 , 4.18]**
Test for overall effect: Z = 0.25 (P = 0.80)				
Heterogeneity: Tau² (DL[b]) = 0.91; Chi² = 8.20, df = 2 (P = 0.02); I² = 76%				
**Total (Wald[a])**			**100.0%**	**0.85 [0.33 , 2.20]**

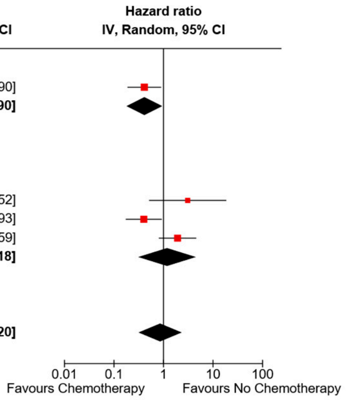

Test for overall effect: Z = 0.33 (P = 0.74)
Test for subgroup differences: Chi² = 1.93, df = 1 (P = 0.16), I² = 48.2%
Heterogeneity: Tau² (DL[b]) = 0.65; Chi² = 11.22, df = 3 (P = 0.01); I² = 73%

Footnotes
[a]CI calculated by Wald-type method.
[b]Tau² calculated by DerSimonian and Laird method.

Figure S8c Local Recurrence Free Survival: Univariate Hazard Ratios (Angiosarcoma Only)

Study or Subgroup	log[HR]	SE	Weight	Hazard ratio IV, Fixed, 95% CI
**8.3.1 Univariate**				
Salminen et al.[a]	0.006976	0.753297	6.4%	1.01 [0.23 , 4.41]
**Subtotal**			**6.4%**	**1.01 [0.23 , 4.41]**
Test for overall effect: Z = 0.01 (P = 0.99)				
Heterogeneity: Not applicable				
**8.3.2 Multivariate**				
Michot et al.[b]	-0.798508	0.222693	73.6%	0.45 [0.29 , 0.70]
Torres et al.[c]	-1.049822	0.427043	20.0%	0.35 [0.15 , 0.81]
**Subtotal**			**93.6%**	**0.43 [0.29 , 0.63]**
Test for overall effect: Z = 4.32 (P < 0.0001)				
Heterogeneity: Chi² = 0.27, df = 1 (P = 0.60); I² = 0%				
**Total**			**100.0%**	**0.45 [0.31 , 0.66]**
Test for overall effect: Z = 4.17 (P < 0.0001)				
Test for subgroup differences: Chi² = 1.22, df = 1 (P = 0.27), I² = 17.9%				
Heterogeneity: Chi² = 1.49, df = 2 (P = 0.47); I² = 0%				

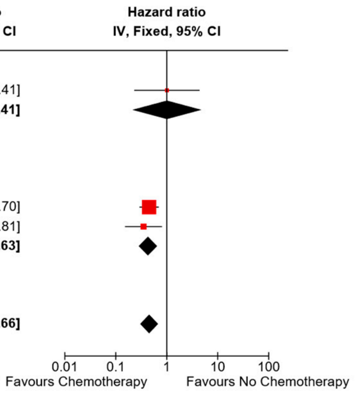

Footnotes
[a]Adjuvant Chemotherapy
[b]Neoadjuvant Chemotherapy
[c]Mixed Chemotherapy(neoadjuvant/adjuvant)

Figure S8d Distant Recurrence Free Survival: Univariate Hazard Ratios (Angiosarcoma Only)

Study or Subgroup	log[HR]	SE	Weight	Hazard ratio IV, Fixed, 95% CI
**8.4.1 Adjuvant Chemotherapy**				
Salminen et al.	0.501987	0.781932	15.0%	1.65 [0.36 , 7.65]
**Subtotal**			**15.0%**	**1.65 [0.36 , 7.65]**
Test for overall effect: Z = 0.64 (P = 0.52)				
Heterogeneity: Not applicable				
**8.4.2 Neoadjuvant Chemotherapy**				
Michot et al.	-0.182362	0.363316	69.6%	0.83 [0.41 , 1.70]
**Subtotal**			**69.6%**	**0.83 [0.41 , 1.70]**
Test for overall effect: Z = 0.50 (P = 0.62)				
Heterogeneity: Not applicable				
**8.4.3 Mixed (neoadjuvant/adjuvant)**				
Cohen-Hallaleh et al.	0.732368	0.772686	15.4%	2.08 [0.46 , 9.46]
**Subtotal**			**15.4%**	**2.08 [0.46 , 9.46]**
Test for overall effect: Z = 0.95 (P = 0.34)				
Heterogeneity: Not applicable				
**Total**			**100.0%**	**1.06 [0.59 , 1.93]**
Test for overall effect: Z = 0.20 (P = 0.84)				
Test for subgroup differences: Chi² = 1.52, df = 2 (P = 0.47), I² = 0%				
Heterogeneity: Chi² = 1.52, df = 2 (P = 0.47); I² = 0%				

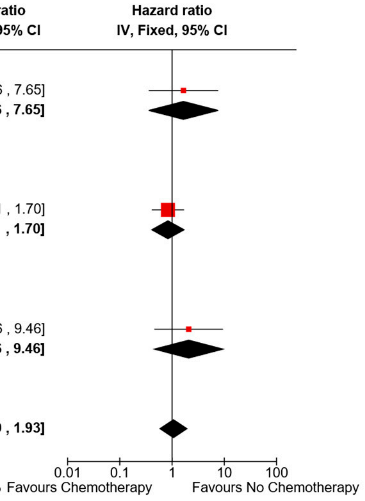

Table S1 Quality assessment of included studies using the Newcastle–Ottawa Scale (NOS) for cohort studies.

**Autor**	**Selection 1**	**Selection 2**	**Selection 3**	**Selection 4**	**Comparability 1**	**Comparability 2**	**Outcome 1**	**Outcome 2**	**Outcome 3**	**Total**
Callesen et al.	Yes	No	Yes	Yes	Yes	No	Yes	Yes	Yes	7/9
Cha et al.	Yes	No	Yes	Yes	Yes	No	Yes	Yes	Yes	7/9
Cohen-Hallaleh et al.	Yes	No	Yes	Yes	Yes	No	Yes	Yes	Yes	7/9
D'Angelo et al.	Yes	No	Yes	Yes	Yes	No	Yes	Yes	Yes	7/9
Feinberg et al.	Yes	Yes	Yes	Yes	Yes	No	Yes	Yes	Yes	8/9
Garayua-Cruz et al.	No	No	Yes	Yes	No	No	Yes	Yes	Yes	5/9
Gladdy et al.	Yes	Yes	Yes	Yes	Yes	Yes	Yes	Yes	Yes	9/9
Guram et al.	Yes	No	Yes	Yes	Yes	No	Yes	Yes	Yes	7/9
Italiano et al.	Yes	No	Yes	Yes	Yes	No	Yes	Yes	Yes	7/9
Joo et al.	No	No	Yes	Yes	No	No	Yes	Yes	Yes	5/9
Kao et al.	Yes	Yes	Yes	Yes	Yes	Yes	Yes	Yes	Yes	9/9
Kuba et al.	Yes	No	Yes	Yes	Yes	Yes	Yes	Yes	Yes	8/9
Lazarides et al.	Yes	Yes	Yes	Yes	Yes	Yes	Yes	Yes	Yes	9/9
Li et al.	Yes	Yes	Yes	Yes	Yes	Yes	Yes	Yes	Yes	9/9
Linthorst et al.	No	No	Yes	Yes	No	No	Yes	Yes	Yes	5/9
Michot et al.	Yes	Yes	Yes	Yes	Yes	Yes	Yes	Yes	Yes	9/9
Mito et al.	Yes	Yes	Yes	Yes	Yes	Yes	Yes	Yes	Yes	9/9
Neuhaus et al.	Yes	No	Yes	Yes	Yes	No	Yes	Yes	Yes	7/9
Palassini et al.	Yes	Yes	Yes	Yes	Yes	Yes	Yes	Yes	Yes	9/9
Patel et al.	No	No	Yes	Yes	No	No	Yes	Yes	No	4/9
Ribeiro et al.	Yes	No	Yes	Yes	Yes	No	Yes	Yes	Yes	7/9
Salminen et al.	Yes	No	Yes	Yes	Yes	No	Yes	Yes	Yes	7/9
Spalek et al.	Yes	No	Yes	Yes	Yes	No	Yes	Yes	Yes	7/9
Torres et al.	Yes	No	Yes	Yes	Yes	No	Yes	Yes	Yes	7/9

Tsuda et al.	Yes	No	Yes	Yes	Yes	No	Yes	Yes	Yes	7/9
van der Burg et al.	Yes	Yes	Yes	Yes	Yes	No	Yes	Yes	Yes	8/9
Wong et al.	Yes	No	Yes	Yes	No	No	Yes	Yes	Yes	6/9
Yin et al.	Yes	Yes	Yes	Yes	Yes	Yes	Yes	Yes	Yes	9/9

Abbreviation: Selection 1: Representativeness of the exposed cohort, Selection 2: Selection of the non-exposed cohort; Selection 3: Ascertainment of exposure; Selection 4: Demonstration that outcome of interest was not present at start of study; Comparability 1: Comparability of cohorts on the basis of the design or analysis controlled for confounders; Comparability 2: Comparability of cohorts on the basis of the design or analysis controlled for confounders; Outcome 1: Assessment of outcome; Outcome 2: Was follow-up long enough for outcomes to occur?; Outcome 3: Indicate the median duration of follow-up and a brief rationale for the assessment above

Table S2 Distribution of tumor grade across included studies and grading systems used

Study	G1	G2	G3	Not assesed	System
Salminen et al.		6	38	6	FNCLCC
Spalek et al.	3	16	39		Not specified
Kao et al.	1	15	54	25	FNCLCC
Michot et al.			69	111 (G1, G2, N/A)	FNCLCC
Ribeiro et al.	10	22	39		FNCLCC
Mito et al.	7	31	51	11	FNCLCC
Italiano et al.	56	175	220	59	Not specified
Yin et al.	13	22	100	38	AJCC 7
Cha et al.	22		84	2	Histology-based grading (Hajdu criteria); high vs low)
Torres et al.	18		54	23	Pathology-based grading (not specified)

G1–G3 denote tumor grade according to the respective grading system used in each study. "Not assessed" includes cases where tumor grade was not reported or could not be classified. Fédération Nationale des Centres de Lutte Contre le Cancer (FNCLCC); American Joint Committee on Cancer, 7th edition (AJCC 7); In studies using dichotomous or grouped grading systems, low-grade tumors were assigned to a combined G1/G2 category and high-grade tumors to G3, without further subdivision.

Table S3 Overview of systemic treatment regimens and agents used across included studies

Study	Regime	Agents
Torres et al.	Mixed Cohort: neoadjuvant/adjuvant	Gemcitabine/Docetaxel (n=13), Paclitaxel (n=12), Doxorubicin/Ifosfamid ± Paclitaxel (n=8), Doxorubicin ± Dacarbazin (n=8), Interferon (n=5), Gemcitabine mono (n=2), other (n=6)
Guram et al.	neoadjuvant	Paclitaxel (n = 10)
Salminen et al.	Adjuvant	Paclitaxel n = 3 or<br>Doxorubicin n = 1 or<br>Docetaxelandfluorouracil/epirubicin/cyclofosfamidea n = 1
Cohen-Hallaleh et al.	Mixed Cohort: neoadjuvant/adjuvant	doxorubicin (n=2) or paclitaxel (n = 5)
Spalek et al.	Perioperative	unspecified (n = 23)
Garayua-Cruz et al.	Mixed Cohort: neoadjuvant/adjuvant	unspecified (n = 5)
Kao et al.	unspecified	unspecified
Palassini et al.	Mixed Cohort: neoadjuvant/adjuvant	Anthracycline-based CT 10/84 (11.9%; 5 combo + ifosfamide, 5 mono); gemcitabine-based CT 33/84 (39.3%; 18 + docetaxel, 15 mono); both regimens 7/84 (8.3%); taxane monotherapy (paclitaxel) 11/84 (13.1%)
Michot et al.	Neoadjuvant	anthracyclines or taxanes; however, detailed information on specific regimens was unavailable n= 38
Van der Burg et al.	Neoadjuvant	paclitaxel (n = 11) or doxorubicin (n = 2)
Wong et al.	Mixed Cohort: neoadjuvant/adjuvant	PLD: Pegylated liposomal doxorubicin (n=6)
Lazarides et al.	Mixed Cohort: neoadjuvant/adjuvant	(Adriamycin+Cisplatin or<br>Ifosfamide+Adriamycin or<br>Etoposide) n = 14 Vs. (Methotrexate+Adriamycin+ Cisplatin) n = 6

Narrative Overview Sex, RIS Localisation, Histopathology

In addition to the poolable HRs for the above-mentioned risk factors, we found other risk factors that cannot be pooled due to different outcomes. Regarding the risk factor sex, two studies reported that no significant effect could be detected with regard to DSS (pelvic and sacral osteosarcomas) (Lazarides et al.) and OS (mixed RIS cohort) (Kao et al). In one study (Ribeiro et al.), female sex and breast as the primary tumor site were associated with improved prognosis in univariate analysis (HR 3.46 [95% CI 1.53–7.83] and HR 2.8 [95% CI 0.9–8.2]), but these associations were not retained after multivariable adjustment. Five studies reported HRs for tumor location (Kao et al., Lazarides et al., Yin et al., Callesen et al., Spalek et al.). Compared with CNS tumors, extremity lesions showed significantly better survival (upper extremity: HR 0.06; [95% CI 0.01–0.71]), and breast tumors also showed a potential advantage (HR 0.09 [95% CI 0.01–1.00]). In contrast, PRS of the pelvis showed a disadvantage compared with lesions of the breast/chest wall (HR 2.3 [95% CI 1.1–4.7]). However, other localisations such as the thorax or abdomen did not differ significantly.

Reconstructive surgery was evaluated in a pooled analysis based on two studies. No significant association with OS was observed (HR 0.55 [95% CI 0.24–1.26]; I^2=0%) (Figure S6.5); however, due to the limited number of studies and small osteosarcoma cohorts, results should be interpreted with caution.

Regarding histopathology, two studies comparing angiosarcoma versus other RIS entities showed no significant association with LRFS (pooled HR 1.81 [95% CI 0.64–5.15]; I^2=54%), although a non-significant trend towards worse outcomes in angiosarcoma was observed (Figure S3.3).

Table S4 Full electronic search strategy

Pubmed	(<br>"sarcoma"[Mesh:noexp] OR<br>Sarcoma*[tiab] OR<br>Angiosarcoma*[tiab] OR<br>"Hemangiosarcoma"[Mesh] OR<br>Hemangiosarcoma*[tiab]<br>)<br>AND (<br>"Neoplasms, Radiation-Induced"[**Mesh**] OR<br>Radiation-induced[tiab] OR<br>Radiation-associated[tiab] OR<br>Post-radiation[tiab] OR<br>Postradiation[tiab] OR<br>"after radiotherapy"[tiab] OR<br>"received radiotherapy"[tiab]<br>)
Embase	('sarcoma'/**de** OR 'sarcoma*':ti,ab,kw OR 'angiosarcoma*':ti,ab,kw OR 'angiosarcoma'/**exp** OR 'hemangiosarcoma*':ti,ab,kw) AND ('radiation induced neoplasm'/**exp** OR 'radiation-induced':ti,ab,kw OR 'radiation-associated':ti,ab,kw OR 'post-radiation':ti,ab,kw OR 'postradiation':ti,ab,kw OR 'after radiotherapy':ti,ab,kw OR ' received radiotherapy':ti,ab,kw)

Cochrane Library	(<br>Sarcoma*:ti,ab,kw OR<br>Angiosarcoma*:ti,ab,kw OR<br>Hemangiosarcoma*:ti,ab,kw<br>)<br>AND (<br>Radiation-induced:ti,ab,kw OR<br>Radiation-associated:ti,ab,kw OR<br>post-radiation:ti,ab,kw OR<br>postradiation:ti,ab,kw OR<br>"after radiotherapy":ti,ab,kw OR<br>"received radiotherapy":ti,ab,kw<br>)

Table S5 PRISMA 2020 Checklist

Topic	No.	Item	Location where item is reported
**TITLE**			
**Title**	1	Identify the report as a systematic review.	Page 1
**ABSTRACT**			
**Abstract**	2	See the PRISMA 2020 for Abstracts checklist	
**INTRODUCTION**			
**Rationale**	3	Describe the rationale for the review in the context of existing knowledge.	Page 3
**Objectives**	4	Provide an explicit statement of the objective(s) or question(s) the review addresses.	Page 3
**METHODS**			
**Eligibility criteria**	5	Specify the inclusion and exclusion criteria for the review and how studies were grouped for the syntheses.	Page 4
**Information sources**	6	Specify all databases, registers, websites, organisations, reference lists and other sources searched or consulted to identify studies. Specify the date when each source was last searched or consulted.	Page 4
**Search strategy**	7	Present the full search strategies for all databases, registers and websites, including any filters and limits used.	Supplementary
**Selection process**	8	Specify the methods used to decide whether a study met the inclusion criteria of the review, including how many reviewers screened each record and each report retrieved, whether they worked independently, and if applicable, details of automation tools used in the process.	Page 4
**Data collection process**	9	Specify the methods used to collect data from reports, including how many reviewers collected data from each report, whether they worked independently, any processes for obtaining or confirming data from study investigators, and if applicable, details of automation tools used in the process.	Page 4-5

Topic	No.	Item	Location where item is reported
**Data items**	10a	List and define all outcomes for which data were sought. Specify whether all results that were compatible with each outcome domain in each study were sought (e.g. for all measures, time points, analyses), and if not, the methods used to decide which results to collect.	Page 4-5
	10b	List and define all other variables for which data were sought (e.g. participant and intervention characteristics, funding sources). Describe any assumptions made about any missing or unclear information.	Page 4-5
**Study risk of bias assessment**	11	Specify the methods used to assess risk of bias in the included studies, including details of the tool(s) used, how many reviewers assessed each study and whether they worked independently, and if applicable, details of automation tools used in the process.	Page 4-5
**Effect measures**	12	Specify for each outcome the effect measure(s) (e.g. risk ratio, mean difference) used in the synthesis or presentation of results.	Page 4-5
**Synthesis methods**	13a	Describe the processes used to decide which studies were eligible for each synthesis (e.g. tabulating the study intervention characteristics and comparing against the planned groups for each synthesis (item 5)).	Page 4
	13b	Describe any methods required to prepare the data for presentation or synthesis, such as handling of missing summary statistics, or data conversions.	Page 4
	13c	Describe any methods used to tabulate or visually display results of individual studies and syntheses.	Page 4
	13d	Describe any methods used to synthesize results and provide a rationale for the choice(s). If meta-analysis was performed, describe the model(s), method(s) to identify the presence and extent of statistical heterogeneity, and software package(s) used.	Page 4-5
	13e	Describe any methods used to explore possible causes of heterogeneity among study results (e.g. subgroup analysis, meta-regression).	Page 4-5
	13f	Describe any sensitivity analyses conducted to assess robustness of the synthesized results.	Page 5
**Reporting bias assessment**	14	Describe any methods used to assess risk of bias due to missing results in a synthesis (arising from reporting biases).	N/A
**Certainty assessment**	15	Describe any methods used to assess certainty (or confidence) in the body of evidence for an outcome.	N/A

Topic	No.	Item	Location where item is reported
**RESULTS**			
**Study selection**	16a	Describe the results of the search and selection process, from the number of records identified in the search to the number of studies included in the review, ideally using a flow diagram.	Page 6
	16b	Cite studies that might appear to meet the inclusion criteria, but which were excluded, and explain why they were excluded.	PRISMA Chart
**Study characteristics**	17	Cite each included study and present its characteristics.	Table 1
**Risk of bias in studies**	18	Present assessments of risk of bias for each included study.	Table 1
**Results of individual studies**	19	For all outcomes, present, for each study: (a) summary statistics for each group (where appropriate) and (b) an effect estimate and its precision (e.g. confidence/credible interval), ideally using structured tables or plots.	Table 1-3 & Supplement Table 1-3
**Results of syntheses**	20a	For each synthesis, briefly summarise the characteristics and risk of bias among contributing studies.	Page 6-7
	20b	Present results of all statistical syntheses conducted. If meta-analysis was done, present for each the summary estimate and its precision (e.g. confidence/credible interval) and measures of statistical heterogeneity. If comparing groups, describe the direction of the effect.	Tabl. 2-3, Figure 4, Supplement
	20c	Present results of all investigations of possible causes of heterogeneity among study results.	N/A
	20d	Present results of all sensitivity analyses conducted to assess the robustness of the synthesized results.	Table 2
**Reporting biases**	21	Present assessments of risk of bias due to missing results (arising from reporting biases) for each synthesis assessed.	N/A
**Certainty of evidence**	22	Present assessments of certainty (or confidence) in the body of evidence for each outcome assessed.	N/A
**DISCUSSION**			
**Discussion**	23a	Provide a general interpretation of the results in the context of other evidence.	Page 16-17
	23b	Discuss any limitations of the evidence included in the review.	Page 16-17

Topic	No.	Item	Location where item is reported
	23c	Discuss any limitations of the review processes used.	Page 16-17
	23d	Discuss implications of the results for practice, policy, and future research.	Page 16-17
**OTHER INFORMATION**			
**Registration and protocol**	24a	Provide registration information for the review, including register name and registration number, or state that the review was not registered.	Page 3
	24b	Indicate where the review protocol can be accessed, or state that a protocol was not prepared.	Page 3
	24c	Describe and explain any amendments to information provided at registration or in the protocol.	N/A
**Support**	25	Describe sources of financial or non-financial support for the review, and the role of the funders or sponsors in the review.	Page 5 & 18
**Competing interests**	26	Declare any competing interests of review authors.	Page 18
**Availability of data, code and other materials**	27	Report which of the following are publicly available and where they can be found: template data collection forms; data extracted from included studies; data used for all analyses; analytic code; any other materials used in the review.	Page 18

Table S6 Subgroup Analysis of prognostic factors in post-radiation sarcoma

Risk factor	Effect	Random/ Fixed Model	Subgroup	OS HR [95%CI]	I²	P Value Heterogeneity	Random/ Fixed Model	DSS HR [95%CI]	I²	P Value Heterogeneity
Age	HR>1 favours younger age	Random	Overall effect	1.85 [1.28 , 2.69]	58%	0.02	Fixed	1.16 [1.07 , 1.27]	46%	0.12
			Age > 40					2.60 [0.76 , 8.88]	N/A	N/A
			Age >55					1.75 [0.88 , 3.48]	N/A	N/A
			Age ≥ 60	1.54 [0.97 , 2.45]	60%	0.04				
			AGE ≥65					2.63 [1.14 , 6.06]	0%	0.48
			Age ≥ 70	2.57 [1.60 , 4.11]	12%	0.32		1.14 [1.04 , 1.24]	N/A	N/A
			Age continuous	1.02 [0.98 , 1.07]	69%	0.04				
Tumor size	HR >1 favours smaller Size	Random	Overall effect	1.54 [1.02 , 2.35]	73%	0.002	Fixed	2.06 [1.34 , 3.18]	24%	0.26
			Size ≥ 4.5 cm	2.06 [1.24 , 3.42]	N/A	N/A				
			Size ≥ 5-5.5cm	1.56 [0.52 , 4.67]	70%	0.04		2.42 [1.39 , 4.19]	40%	0.19
			Size ≥ 6.5 cm	3.23 [0.48 , 21.77]	N/A	N/A				
			Size ≥ 10-11 cm					1.60 [0.79 , 3.22]	9%	0.29
			Largest Tumor Diameter 4.7-7.2 cm	1.09 [1.02 , 1.17]	N/A	N/A				
	HR >1 favours smaller Size	Fixed	Size continuous	1.13 [1.05 , 1.21]	46%	0.16				
Grading	HR>1 favours lower Grading	Fixed	Overall effect	2.38 [1.86 , 3.04]	0%	0.46				
			G1 vs G2	1.83 [0.94 , 3.55]	0%	0.46				
			G1 vs G3	3.00 [1.49 , 6.07]	4%	0.35				
			G2 vs G3	4.37 [2.26 , 8.46]	0%	0.47				
			G1/G2 vs G3	2.10 [1.53 , 2.88]	0%	0.69				
Multifocal disease versus single lesion	HR< 1 favours single lesion	Fixed	Overall effect	1.01 [0.70 , 1.45]	0%	0.96				
Local Expertise	HR <1 favours sarcoma center		Overall effect				Fixed	0.27 [0.14 , 0.53]	0%	0.46
Margin	HR >1 favours negative margin	Fixed	Overall effect	2.22 [1.75 , 2.80]	27%	0.2	Fixed	2.13 [1.45 , 3.13]	16%	0.31
			R0 vs R1	1.88 [1.41 , 2.50]	47%	0.13				
			R0 vs R2	3.15 [1.75 , 5.66]	0%	0.8				
			negative vs positive	3.03 [1.74 , 5.29]	0%	0.45				
Resection strategy radical vs. limited	HR<1 favours radical surgery	Random	Overall effect	0.54 [0.23 , 1.22]	57%	0.07	Fixed	0.53 [0.3 , 0.94]	20%	0.29
Radiotherapy for PRS at any time	HR<1 favours Radiotherapy	Fixed	Overall effect	1.37 [0.94 , 1.98]	32%	0.21				
			Adjuvant Radiotherapy	1.91 [1.11 , 3.29]	0%	0.32				
			Mixed (neoadjuvant/adjuvant/unspecified)	1.02 [0.62 , 1.70]	10%	0.33				
Chemotherapy for PRS at any time	HR <1 favours Chemotherapy	Fixed	Overall effect	0.82 [0.60 , 1.12]	31%	0.18	Random	0.85 [0.33 , 2.20]	73%	0.01
			Adjuvant Chemotherapy	2.17 [0.60 , 7.80]	N/A	N/A				
			Mixed (neoadjuvant/adjuvant/unspecified)	0.88 [0.58 , 1.32]	0%	0.52		1.18 [0.33 , 4.18]	76%	0.02
			Neoadjuvant Chemotherapy	0.65 [0.39 , 1.07]	73%	0.05		0.41 [0.19 , 0.90]	N/A	N/A
Surgery vs. No Surgery	HR>1 favours surgery	Random	Overall effect	4.37 [1.34 , 14.25]	88%	< 0.0001				

Abbreviations: overall survival (OS), disease specific survival (DSS), Hazard ratio (HR), HR [95%Confidence Interval], post-radiation sarcoma (PRS), not applicable (N/A)
